# Fundamentals of bio-based technologies for selective metal recovery from bio-leachates and liquid waste streams

**DOI:** 10.3389/fbioe.2024.1528992

**Published:** 2025-01-09

**Authors:** Anna Sieber, Sabine Spiess, Wadih Y. Rassy, Dominik Schild, Thomas Rieß, Shalini Singh, Rohan Jain, Nora Schönberger, Franziska Lederer, Klemens Kremser, Georg M. Guebitz

**Affiliations:** ^1^ K1-MET GmbH, Linz, Austria; ^2^ Department of Science and Technology, Institute of Biotechnology, IMC University of Applied Sciences, Krems, Austria; ^3^ Faculty of Technical Chemistry, TU Wien, Vienna, Austria; ^4^ Helmholtz-Zentrum Dresden-Rossendorf, Helmholtz Institute Freiberg for Resource Technology, Biotechnology Department, Dresden, Germany; ^5^ Department of Agrobiotechnology, IFA-Tulln, Institute of Environmental Biotechnology, BOKU University of Natural Resources and Life Sciences Vienna, Tulln an der Donau, Austria; ^6^ Austrian Centre of Industrial Biotechnology, Tulln an der Donau, Austria

**Keywords:** selective metal recovery, secondary resources, bio-based recovery technologies, bioaccumulation, bioelectrochemical systems, biosorption, metal-binding peptides, siderophores

## Abstract

The number of metal-containing waste streams resulting from electronic end-of life products, metallurgical by-products, and mine tailings to name but a few, is increasing worldwide. In recent decades, the potential to exploit these waste streams as valuable secondary resources to meet the high demand of critical and economically important raw materials has become more prominent. In this review, fundamental principles of bio-based metal recovery technologies are discussed focusing on microbial metabolism-dependent and metabolism-independent mechanisms as sustainable alternatives to conventional chemical metal recovery methods. In contrast to previous reviews which have partially addressed this topic, a special focus will be given on how fundamental principles of bio-based recovery technologies can influence the selectivity and specificity of metal recovery. While conventional methods for metal recovery show benefits in terms of economic affordability, bio-based recovery technologies offer advantages in terms of efficiency and environmentally friendliness. Modifications and adaptations in the processes of biosorption, bioaccumulation and bioelectrochemical systems are highlighted, further emphasizing the application of metal-binding peptides and siderophores to increase selectivity in the recovery of metals. Single metal solutions or mixtures with a low complexity have been the focus of previous studies and reviews, but this does not reflect the nature of complex industrial effluents. Therefore, key challenges that arise when dealing with complex polymetallic solutions are addressed and the focus is set on optimizing bio-based technologies to recover metals efficiently and selectively from bio-leachates or liquid waste streams.

## 1 Introduction

Factors such as limited availabilities and decreasing ore grades lead to a high demand of critical raw materials (CRMs). Additionally, the increasing consumption of such metals due to electrification of the automotive sector and the need to transition to green and sustainable energy technologies drives the demand on metals and minerals even further (Jones et al., 2020; Moe, 2020; Pommeret et al., 2022). The European Union (EU) has therefore declared a list of critical raw materials and materials with relatively high economic importance which has been updated recently (European Commission, 2023b; 2023a). With these factors in mind, the transition to exploiting different waste streams as secondary resources for urban mining approaches has become increasingly prominent within the last few decades. Transition metals, rare earth elements (REEs) and precious metals (PMs) are present in various waste streams and industrial by-products such as waste electronic and electric equipment (WEEE) (Efstratiadis and Michailidis, 2022; Ramprasad et al., 2022), ashes, slags, and dusts from metallurgical industries, waste and coal incineration (Lima et al., 2022), as well as low-grade mine tailings (Šajn et al., 2022; Sarker et al., 2022), to name only a few. While the biological treatment and solubilization of the afore mentioned waste streams in the form of bioleaching is well studied and started to be scaled up from laboratory to pilot and industrial scale (Gericke et al., 2023; Tezyapar Kara et al., 2023; Vera Véliz et al., 2023), the selective recovery of solubilized metals from resulting polymetallic waste streams using different bio-based recovery technologies remains challenging. Numerous studies have investigated the application of bio-based recovery technologies like biosorption (Stathatou et al., 2022), bioprecipitation (Khadim et al., 2019), bioaccumulation (Diep et al., 2018), bioelectrochemistry (Kabutey et al., 2019), the use of metal-binding peptides (Braun et al., 2018), or siderophores (Jain et al., 2019) to recover metals from single-metallic or low metal complexity solutions. Conventional metal recovery technologies such as chemical precipitation, solvent extraction or ion exchange provide benefits such as a reduction in process time, economic affordability, and good selectivity for certain metals, but similarly face the disadvantages of low efficiencies particularly at low target metal concentrations, generation of toxic by-products, and high costs (Taghvaie Nakhjiri et al., 2022). Bio-based metal recovery technologies on the other hand are reported to be cost efficient, provide high recovery efficiencies for certain metals and are environmentally friendly (Ike et al., 2017; Sethurajan et al., 2018). Nevertheless, selectivity and specificity are lower compared to conventional techniques but are known to be crucial parameters for the treatment of complex polymetallic solutions. Studies dealing with the optimization of these parameters are limited. Understanding the fundamental principles behind the different nature-based recovery technologies is therefore essential to increase selectivity and sensitivity. Depending on whether the bio-based recovery process is microbial metabolism-dependent (i.e., bioaccumulation, or bioelectrochemistry) or metabolism-independent (i.e., biosorption, metal-binding peptides, or siderophores), selectivity and specificity are strongly influenced by physical, chemical, and metabolic parameters (Priya et al., 2022).

**FIGURE 1:**
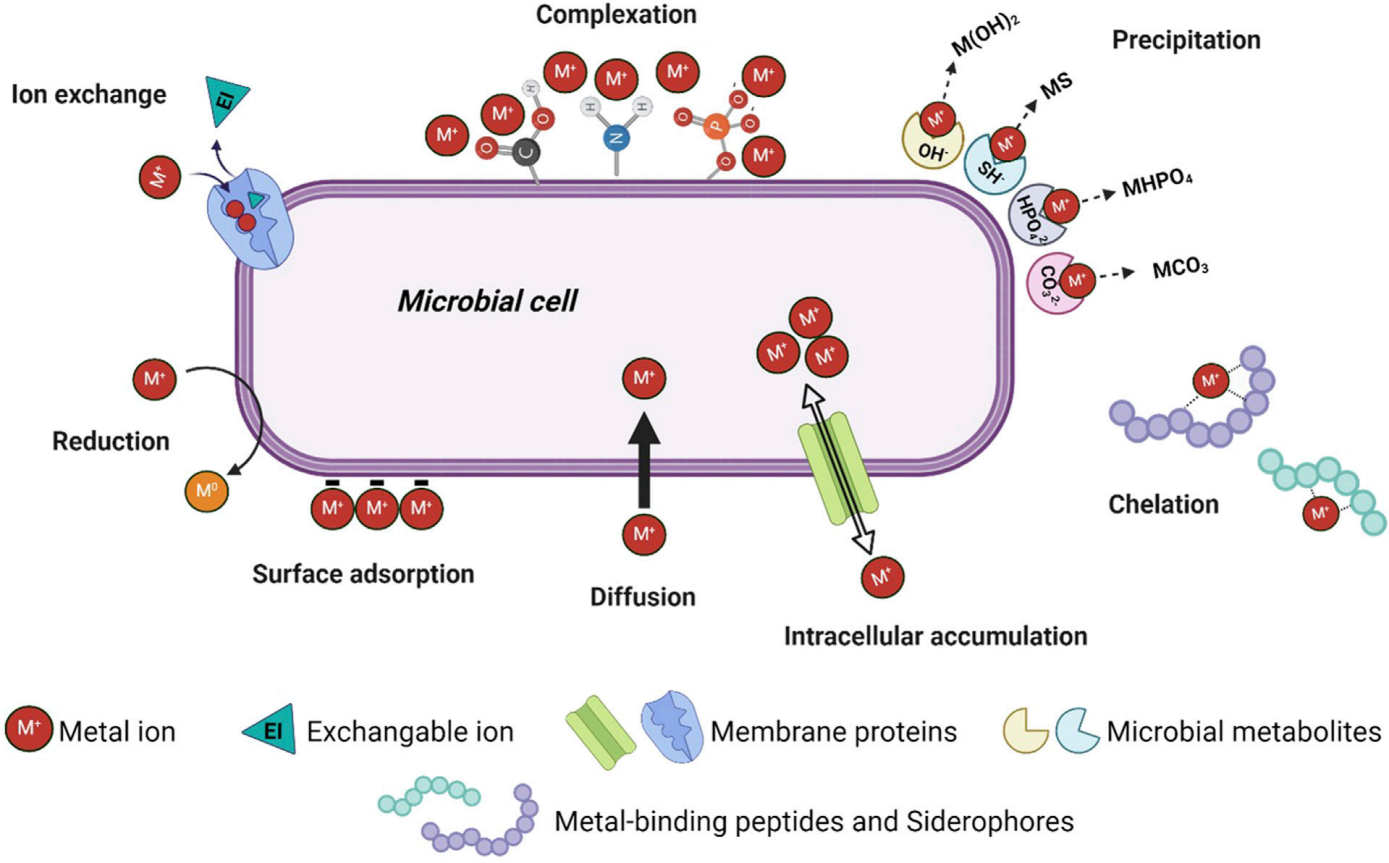
Schematic overview of the most important mechanisms behind the presented bio-recovery methods (Created in BioRender. Kremser, K. (2024) https://BioRender.com/r56w339).

By understanding and investigating the these principles, pathways and factors influencing the different bio-based recovery technologies, current methods can be adapted, and metals can be recovered both selectively and specifically. The aim of the present review is therefore to shed light on the individual bio-recovery technologies, focusing on the fundamental functions and pathways involved in metal recovery (Figure 1) and how changes of these can be beneficial to increase the selectivity and specificity. Recent studies and reviews have focused on a comparison of different technologies, recovery of certain metals, and effectivities of different nature-based processes dealing with single-metal solutions or low complex mixtures (Brown et al., 2023; Shekhar Samanta et al., 2023). In contrast, industrial effluents, acid mine drainage or bioleaching lixiviants present complex waste streams consisting of multi-metal mixtures. Therefore, the present study will focus on polymetallic waste streams and highlight the potential of bio-based recovery technologies for their treatment. A special focus will be given to the difference in metabolism-dependent and metabolism-independent processes and how the fundamental principles behind can be used to increase the selectivity and sensitivity towards complex multi-metal streams. To the best of the authors knowledge, this has not been covered by previous reviews in this field.

## 2 Metabolism-dependent bio-recovery techniques

### 2.1 Bioaccumulation

Bioaccumulation is a key environmental process in which living organisms accumulate chemicals from their environment faster than they are excreted or metabolically degraded. This phenomenon is of particular importance in the context of pollutant management, as it utilizes the natural abilities of various microorganisms to bind and concentrate pollutants, including heavy metals and rare earth elements (REE), from ecosystems (Gupta et al., 2009; Santoro et al., 2009; Chojnacka, 2010).

#### 2.1.1 Fundamentals and mechanisms behind bioaccumulation

Bioaccumulation involves the uptake of substances, that are then accumulated within the cells, organs, or tissues. This accumulation controls the health and development of individuals as well as changing wider ecosystem dynamics (Proc et al., 2021; Nnaji et al., 2023). Therefore, to understand the peculiarities of the functions and consequences, the specific systematics need to be understood in order to guarantee that the approaches are beneficial to ecosystem wellbeing and environmental protection (Gupta et al., 2009; Santoro et al., 2009; Chojnacka, 2010). Additionally, the literature about the mechanics of bioaccumulation seems to be biased towards research in plants and agriculture. There is a need for differentiation between bioconcentration, bioaccumulation, and biosorption to comprehend how intricate these phenomena are. Bioconcentration generally describes the transfer of a water-borne chemical to an aquatic organism. Biosorption, which is described in more detail in Chapter 3.1, specifically involves binding of molecules such as pollutants to surface structures or organisms, whereas bioaccumulation involves all forms of uptake and active transport of substances across cell membranes into the cytosol. This reflects the intricate and multifaceted relationship between organisms and their environments, showing off the complexity of bioaccumulation (Kaduková and Virčíková, 2005; Chojnacka, 2010). The intricate relationship can be modelled to target specific metal pollutants. For example, *Escherichia coli* has been shown to take in REEs and heavy metals in varying concentrations (Table 1), establishing possible industrial uses (Deng and Wilson, 2001; Diep et al., 2018; Rassy et al., 2023). Whereas bioaccumulation can be exploited for various purposes, research focuses on the removal of heavy metal pollutants in both soil and water as well as on the enrichment of various metals, including rare earth elements that are becoming more and more important with the rising use of electronic devices, especially in the context of renewable energy and sustainable technologies (Gupta et al., 2009; Santoro et al., 2009; Matache et al., 2013; Vander Hoogerstraete et al., 2013; Freiderich et al., 2015).

**TABLE 1:** Removal/recovery efficiencies of various metals with bioaccumulation.

Target metal	Source	Biosorbent	Max. recovery [mg g^−1^ or %]	pH	References
La, Nd, Eu, Dy, Yb, Y (and other rare earth metals)	E-waste and wastewater	*Escherichia coli* K12	Up to 53.12% of total REE concentration (2.25 ppm)	6.7–6.8	Rassy et al. (2023)
Tl, As	Soil remediation in mining areas	Pteris vittata (Chinese Brake Fern)	Tl: 0.007–0.111 mg g^-1^, As: 7.22–11.11 mg g^-1^	-	Wei et al. (2020)
Cu, Pb, Cd, As, Hg (and other heavy metals)	Absorption of hazardous pollutants, especially near industrial sites	*Blechnum orientale* (Oriental Blechnum Fern)	Pb: 0.066 mg g^-1^, As: 0.006 mg g^-1^, Hg: 0.0004 mg g^-1^, Cd: 0.008 mg g^-1^, Cu: 0.022 mg g^-1^	-	Zhu et al. (2013)
Ln	Natural and artificial REE sources	*Methylacidiphilum fumariolicum* strain SolV	Up to 47.4% of total Ln concentration	2–3	Singer et al. (2023)
Hg	Wastewater	*E. coli* JM109	26.8 mg g^-1^	9.6	Deng and Wilson (2001)
Cd, Co, Cu, Hg, Ni, U, As	Wastewater	*E. coli* (Various Strains)	Up to 178.72 mg g^-1^ Hg, 60 mg g^-1^ Ni, 4.8 mg g^-1^ Co, 1.25 mg g^-1^ As^3+^, 63.26 mg g^-1^ Cd, 145 mg g^-1^ Cu, 10700 mg g^-1^ U	6–7	Diep et al. (2018)

Moreover, bioaccumulation demonstrates versatility in application since it can be observed in both wild organisms and genetically altered ones as well. Deng et al. (2013) investigated the bioaccumulation of Ni^2+^ in recombinant *E. coli* cells expressing different nickel-affinity transmembrane proteins (NiCoTs) and Metallthionein (MT). The recombinant strain N1c expressing NiCoTs from *Helocobacter pylori* in addition to MT reached a maximum Ni^2+^ uptake capacity of 83.33 mg g^−1^ compared to 35.71 mg g^−1^ for the wild type *E. coli*. confirming that both, NiCoT and MT, are essential for effective Ni^2+^ bioaccumulation (Deng et al., 2013). These research efforts on improving the bioaccumulative abilities of microbes, e.g., by recombinant expression of metal import-storage systems, show a high potential for heavy metal removal and recovery from wastewater effluents (Diep et al., 2018; Nnaji et al., 2023).

The uptake of metals in organisms involves intricate mechanisms influenced by a combination of environmental and biological factors. Metals associated with transport carriers significantly influence the control of their flux across cell membranes. Effective uptake requires that the flux of free metal ions through the diffusion layer at the organism’s surface exceeds the metal uptake flux; otherwise, diffusion toward the cell membrane becomes the rate-limiting step (Hudson, 1998; Kalis et al., 2006).

Furthermore, lipophilic metal-ligand complexes can bypass conventional metal transport routes, crossing biological membranes via passive diffusion. Some organisms produce these ligands to enhance the uptake of essential metals by complexation (Phlnney and Bruland, 1994; Fortin and Campbell, 2000; Kalis et al., 2006). Research suggests that strongly bound metals, like Cu and Pb, to organic matter exhibit slower transport rates compared to weakly bound metals, implying that the release of metals from organic carriers is a potential limiting factor. The lack of observed competition effects between metals for adsorption and uptake in roots suggests that such effects might only become relevant at higher metal concentrations (Weggler et al., 2004).

The biodynamic model, developed by Luoma and Rainbow (2005), offers a robust framework to explain variability in metal bioaccumulation, integrating geochemical influences, biological differences, and species-specific characteristics. It quantifies uptake rates from water and food and calculates loss rate constants, demonstrating that bioaccumulation results from a dynamic balance of these factors. This model provides a systematic method for predicting metal contamination in food chains, allowing for better environmental risk assessment. Additionally, it aids in the development of targeted bioremediation strategies by identifying species with desirable bioaccumulation traits. The model’s alignment with field data highlights the significance of dietary uptake and species-specific physiological processes, providing a unified explanation for observed bioaccumulation patterns across different species and environments (Luoma and Rainbow, 2005).

Additionally, the study by Kalis et al. (2006) examines how humic acid influences the uptake of metals in plants by altering their free and labile concentrations. Specifically, humic acid decreases the adsorption of Cu, Pb, and Fe at root surfaces but increases that of Cd, Zn, and Mn, likely due to the high-affinity complexation of metals with organic matter. This differential effect underscores the competitive interactions between metal ions in multicomponent systems, affecting uptake dynamics (Kalis et al., 2006).

Numerous studies have explored metal enrichment in microorganisms, though the precise mechanisms remain underexplored (Markai et al., 2003; Tsuruta, 2007; Bonificio and Clarke, 2016). Metal uptake occurs either as complexes or in ionic forms through chemical interactions, some of which can be activated or deactivated by environmental conditions (Cho and Kim, 2003; Hirose, 2022). It is hypothesized that transmembrane ion intake occurs via transporter molecules, which can relocate ionic or complexed metals, thus maintaining metal ion homeostasis (Theodoulou and Kerr, 2015; Mandal et al., 2019).

#### 2.1.2 Advantages, limitations and overcoming bottlenecks

Metal uptake mechanisms in organisms are complex networks influenced by various factors such as metal speciation changes, the presence of metal transporters, antioxidant responses, endocytic processes, and metal tolerance mechanisms (Wang et al., 1996; Azevedo et al., 2007). Understanding these processes requires consideration of metal food sources, bioavailability, and environmental impacts on metal accumulation (Amiard-Triquet and Amiard, 1998; Ahlf et al., 2009; Stewart et al., 2015). Identifying specific transporters, such as NRAMP1 and NRAMP5 in Arabidopsis for manganese, iron, and cadmium transport, is crucial for delineating specific uptake pathways (Ishimaru et al., 2012; Agorio et al., 2017). The interaction of metals with secondary messenger systems and the resultant oxidative stress is a common pathway affecting all organisms (Rengel, 2004). Trace metal movement along food chains and their bioaccumulation in aquatic organisms like mussels further illustrates the complex dynamics of metal uptake (Fisher et al., 1996).

The role of metals in living organisms is dual-faceted; while certain metals are essential, excessive levels can be toxic, adversely affecting overall health (Davidov et al., 2019; Dahiya and Dahiya, 2022). The impact of metal exposure on animal and plant health is significant, often leading to detrimental effects (Bashir et al., 2014). Besides, hydrometallurgical metal recovery methods often rely on acids such as H_2_SO_4_, HCl or HNO_3_ since metal ions are easily solubilized under acidic conditions. Hence, extremophilic bacteria that strive at low pH and can tolerate high concentrations of toxic elements (e.g., Hg, Fe, As, Se and U) hold great potential for recovery of metals by bioaccumulation (Singer et al., 2023). Integrating bioaccumulation with other methods, such as bioleaching, can enhance metal extraction and concentration, offering a promising avenue for bioremediation and resource recovery (Rassy et al., 2023; Lalropuia et al., 2024).

The interplay between metal transporters, ligand complexes, and environmental conditions reveals the complexity of metal uptake pathways, highlighting the need to understand these dynamics for improved pollutant management (Hudson, 1998; Kalis et al., 2006). Future applications, such as the removal of metals from wastewater and soil using plants and microorganisms, demonstrate the versatility and adaptability of bioaccumulative processes in both natural and engineered systems (Zhu et al., 2013; Thompson and Vaughan, 2014; Wei et al., 2020). As research continues to explore these mechanisms, bioaccumulation will remain one of the most valuable tools in the pursuit of sustainable environmental remediation and pollutant management (Rassy et al., 2023; Lalropuia et al., 2024).

#### 2.1.3 Application of bioaccumulation in bio-leachates and liquid waste streams

Methylotrophic bacteria have been shown to incorporate light lanthanides into protein structures, although the precise uptake mechanisms remain unidentified. XoxF enzymes, highly enriched with light lanthanides from the surrounding soil, have been identified in these bacteria (Roszczenko-Jasińska et al., 2021). In a recent study, Singer and colleagues (2023) used the extremophilic bacterium *Methylacidiphilum fumariolicum* strain SolV to accumulate and enrich certain lanthanides (Ln) from various natural and artificial REE sources. In a large-scale approach, SolV removed up to 47.7% of Ln from 3.7 L of Königstein water derived from a former uranium ore mine after sulfuric leaching. The authors describe a certain selectivity of Ln uptake where at high concentrations (in µM range) the light Ln are accumulated but at low concentrations (up to 100 nM) the heavier Ln can additionally be removed in selected fractions (Singer et al., 2023). Furthermore, the model organisms *E. coli K12,* isolated more than 100 years ago, also demonstrates the ability to accumulate various lanthanides, with ongoing studies investigating the conditions under which significant uptake of rare earth elements can occur. This knowledge could eventually facilitate the removal of rare earth elements from wastewater or contaminated water sources (Rassy et al., 2023).

The ferns, *Pteris vittate* and *Blechnum orientale* used in phytoremediation of soils could also be used to recover heavy metals from contaminated soils, particularly at mining sites and from bio-leachates. These ferns exhibit a high translocation and bioconcentration factor for metals like As, Tl, and Pb, making them effective in reducing pollution (Zhu et al., 2013; Thompson and Vaughan, 2014; Wei et al., 2020). Microorganisms that bioaccumulate target metals offer another remediation approach, though soil’s complex environment poses challenges, particularly when employing genetically modified strains. This strategy is therefore more frequently applied to liquid environments such as wastewater (Mühlbachová et al., 2005; Kashem et al., 2007; Diep et al., 2018; Danouche et al., 2021). Removal and recovery efficiencies using bioaccumulation with additional organisms are summarized in Table 1.

### 2.2 Bioelectrochemistry

In recent years, the application of bioelectrochemical systems (BES) for the removal and recovery of metals from various aqueous sources, such as heavy metal contaminated wastewaters (Huang et al., 2022), industrial process streams (Li et al., 2008) or (bio-) leachates (Gálvez et al., 2009; Spiess et al., 2023) has attracted increasing research attention. Beside these BES have also been extensively studied for other purposes such as harnessing electricity from wastewater (Liu et al., 2004), generating value-added chemicals by conversion of the greenhouse gas CO_2_ (Ganigué et al., 2015), or water desalination (Kim and Logan, 2013).

#### 2.2.1 Fundamentals and mechanisms behind bioelectrochemical systems

**FIGURE 2:**
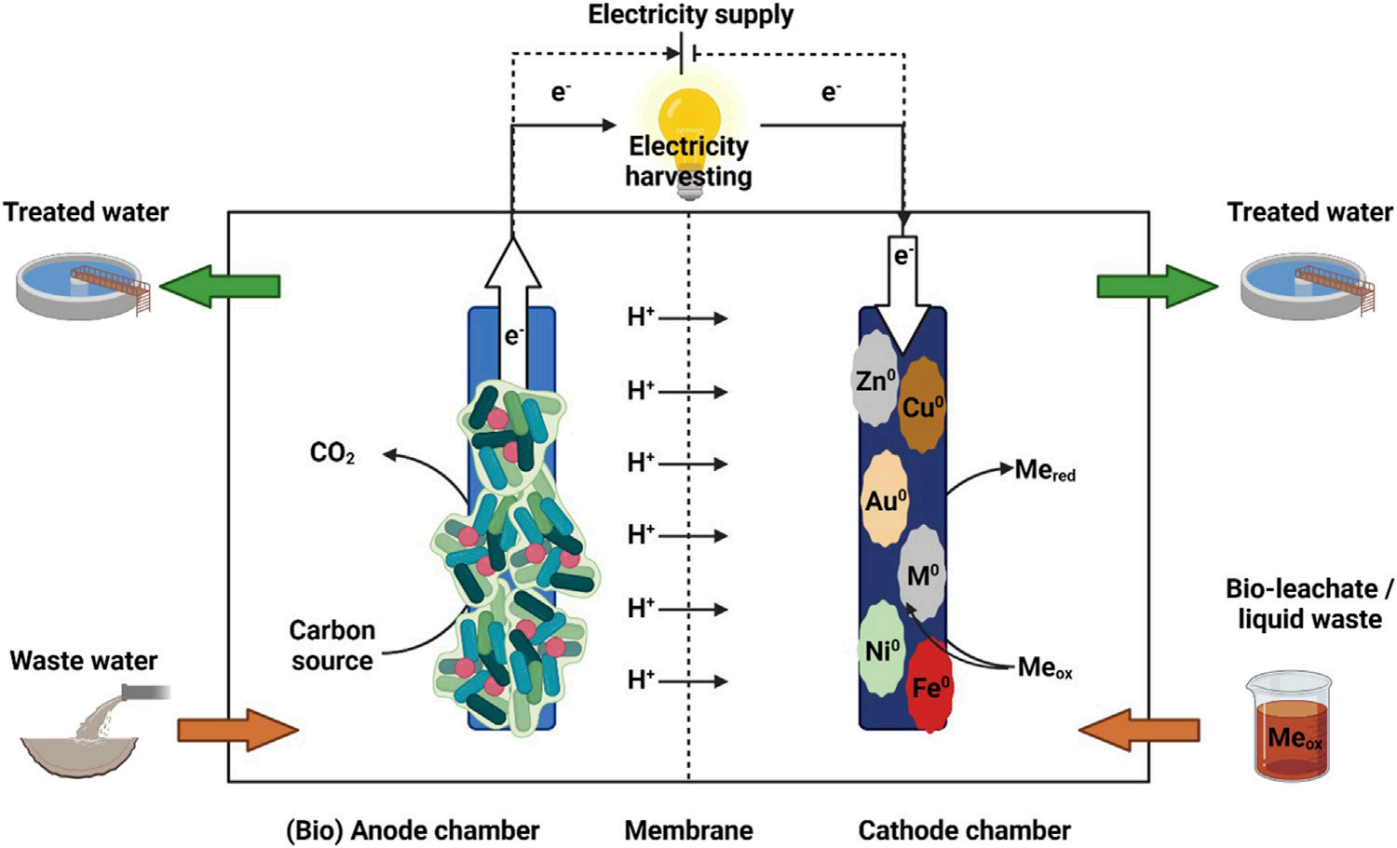
Schematic presentation of a bioelectrochemical system (BES) (Created in BioRender. Kremser, K (2025) https://BioRender.com/a18w131).

BES merge microbial and electrochemical processes to convert chemical energy into electrical energy and *vice versa*. BES consist of an anode, where oxidation processes take place, and a cathode, where reduction processes occur, and are typically separated by a membrane (Figure 2). The fluid, containing the reactants or products is referred as electrolyte, or more precisely as anolyte or catholyte, depending on the related electrode (Rabaey and Rozendal, 2010). In BES, microorganisms are either used to generate electrons by degrading organic matter and transferring them to a solid electrode, which serves as the electron acceptor, or they can take up electrons from the electrode for product formation (Rabaey and Rozendal, 2010). Electroactive microorganisms can interact with the electrodes via direct or mediated/indirect electron transfer mechanisms (Harnisch and Rabaey, 2012; Logan et al., 2019). For direct electron transfer mechanism outer membrane proteins, such as cytochromes, or conductive extensions (known as pili or nanowires) are used for electron transfer (Rosenbaum et al., 2011). Indirect transfer uses externally added or self-produced mediators (e.g., flavin) to shuttle electrons between the electrode and the microbe (Kumar et al., 2016). There is also evidence for direct interspecies electron transfer between the same species, genera, or even between different phyla (Logan et al., 2019). Depending on power production or power investment, BES can be classified as a microbial fuel cell (MFC) or as a microbial electrolysis cell (MEC) (Rabaey and Rozendal, 2010). In general, the microbial degradation of organic compounds at the anode produces electrons which are used at the cathode to drive aseptic metal reduction reactions or metal precipitation (Nancharaiah et al., 2015). However, the use of biocathodes for metal recovery from very low concentrated liquid waste streams is also possible, as demonstrated by microbial catalysis with Co(II) reduction with simultaneous methane and acetate production in a MEC dominated by species such as *Geobacter psychrophilus* and *Acidovorax ebreus* (Huang et al., 2014), and also for Cr(VI) reduction to Cr(III) assisted by *Trichococcus pasteurii* and *Pseudomonas aeruginosa* (Tandukar et al., 2009). If the redox potential of the cathodic half-cell reaction is either comparable to or higher than the anode potential, generated by microbial oxidation, the net cell voltage is positive and power is generated, while metal recovery takes place spontaneously. Therefore, metal ions with a positive redox potential such as Cu(II), Ag(I), Pd(II), Au(III), Cr(VI), and Co(III) have been successfully recovered in MFC mode. However, if the half-cell reaction is below the anode potential, voltage supply is necessary to drive thermodynamically unfavorable metal reduction (Nancharaiah et al., 2016). Thus metal recovery of Ni(II), Cd(II), and Zn(II) can be accomplished in MECs by applying a small external power (Nancharaiah et al., 2015).

#### 2.2.2 Advantages, limitations and overcoming bottlenecks

Compared to electrowinning, BES offer several advantages, 1) no or less energy input is needed to drive metal recovery, 2) simultaneous treatment of wastewater is possible, 3) cheaper anode materials and self-reproducing biocatalysts can be used (Modin et al., 2012; Hemdan et al., 2022). However, despite reaching remarkable metal recovery efficiencies from single and mixed metal solutions, this technology is still at a low technology readiness level since several challenges, such as improving the efficiencies and process stability, use of affordable materials (electrodes and membrane) to reduce the capital costs, economic long-term operation, standardization of performance parameters and a thorough understanding of microbial and electrochemical interactions, need to be tackled before scaling-up this technology. BES can be either scaled-up from lab scale by enlarging the volumes of the working chambers and the electrode surface areas or by connecting multiple modular units to a stacked module (Jadhav et al., 2022). Therefore, further studies are needed using real liquid waste streams or (bio-) leachates as catholytes, focusing on increasing the metal recovery selectivity, and understanding and controlling the factors influencing metal recovery, such as applied voltage, metal ion concentration, retention time, and electrolyte conductivity. Also, pH splitting (namely, a decrease of the anolyte pH and increase of the catholyte pH due to electrochemical reactions) has been reported as an obstacle for metal recovery using BES. On the one hand, an anolyte pH decrease can lead to inhibited microbial processes, as a low pH is not favorable for organic matter oxidation (Zhang et al., 2020). On the other hand, a catholyte pH increase favors the formation of metal hydroxide complexes such as Me(OH)^+^ or Me(OH)_2_ and affects electrochemical reactions, whereas a very low catholyte pH leads to a competition of H_2_ evolution with metal reduction (Modin et al., 2017). Furthermore, low electricity is produced due to limited capacity of electroactive microbes, leading to longer reaction times compared to electrowinning (Zhang et al., 2020). However, in recent years the modelling of bioelectrochemical systems combined with machine learning attracted research attention as a valuable model to improve the understanding of limitations and predicting the electricity generation (Zakir Hossain et al., 2023). Also, the application of biocathodes has been successfully demonstrated using solutions with low metal concentrations, but at higher concentrations microbial activity was suppressed, therefore species with higher metal tolerance need to be identified (Wang and Ren, 2014).

#### 2.2.3 Application of bioelectrochemistry in bio-leachates and liquid waste streams

First, microbial electrochemical technology was mainly used for metal removal from contaminated wastewater. For example, Wang et al. (2008) demonstrated the reduction of mutagenic and carcinogenic Cr(VI) to non-toxic Cr(III) using synthetic Cr(VI) containing wastewater as MFC catholyte and anaerobic microorganisms as anodic biocatalysts. Hereafter, Heijne et al. (2010) demonstrated as first, the application of MFCs for the purpose of metal recovery. Cu was recovered from a low pH copper chloride solution on a graphite foil cathode with an efficiency of >99.88% while producing a current density of 3.2 A m^−2^ in a flow channel MFC, separated by a bipolar membrane (Heijne et al., 2010). Subsequently, research on metal recovery from single metal solutions has been expanded to simulated multi-metal solutions, or even real wastewater and bio-leachate. For example, selective metal recovery of Cu(II), Pb(II), Cd(II) and Zn(II) from a mixed metal solution, simulating a municipal solid waste fly ash leachate, has been demonstrated for the first time in a two-compartment cell separated by an anion exchange membrane (Modin et al., 2012). The cell was first operated in MFC mode to recover Cu, then Pb was recovered by controlling the cathode at a potential of −0.51 V vs. standard hydrogen electrode (SHE), next Cd was recovered by applying −0.66 V vs. SHE on the cathode, and afterwards the anode was controlled at +0.2 V vs. SHE to deposit Zn onto a titanium wire. In the first period 99.9% of the metals deposited onto the cathode accounted for Cu the rest were trace amounts of Pb. In the second period 92.7% Pb and 7.3% of Cu, in the third period 82.3% Cd, 16% Pb and 1.7% Cu, and in period four 100% Zn have been deposited onto the cathode surface (Modin et al., 2012). Another study investigated the removal of the heavy metals Fe(II), Ni(II), and Cu(II) from artificial acid mine drainage at pH 2.85 using a MEC. The MEC was operated in fed-batch mode by applying a fixed voltage of 1 V. First, Cu was recovered in its elemental form, followed by Ni and finally Fe, suggesting that a selective recovery of metals could be achieved due to different metal reduction potentials and by controlling the hydraulic catholyte retention time. However, recovery delays have been noticed when comparing the mixed metal solution with the single metal one, probably due to the competition for electrons among protons and metal ions (Luo et al., 2014). In addition, Co recovery from stripping solution of spent lithium ion-batteries by precipitation of Co(II) as Co(OH)_2_ and CoCO_3_, by increasing the catholyte pH from 4 to >8, has been demonstrated using a MFC (Huang et al., 2019a). Furthermore, to reduce the external energy consumption, power obtained from a MFC for Cu recovery was used to power a MEC for elemental Co recovery, resulting in 65.3%–72% Co recovery (Wu et al., 2015). Another study investigated the selective recovery of Zn and Pb from mining wastewater (Zhang et al., 2020). Initially, 98.5% ± 1.4% Pb was recovered at a cathode potential of −0.75 V vs. Ag/AgCl within 10 h of BES operation, afterwards the cathode potential was increased to −1.2 V vs. Ag/AgCl and 98.7% ± 0.7% Zn recovery was obtained (Zhang et al., 2020). The use of various multi metal solutions as BES catholyte has been further expanded to real bio-leachate. Zn was deposited onto the cathode surface of a MEC by applying −100 mV vs. Ag/AgCl on a bioanode, achieving a Zn recovery efficiency of 41% ± 13% and an energy consumption of 2.55 kWh kg^−1^ (Spiess et al., 2023). The possibility of combining microbial electrochemical technology with bioleaching has also been demonstrated, as simultaneous copper leaching and electricity generation from chalcopyrite concentrate was feasible. Therefore, an anode, to oxidize reduced sulfur compounds to sulfuric acid, was implemented into the deep mineral layer of a bioleaching column, while the cathode was fixed in the upper solution (Huang et al., 2019b). Recently, for the first-time simultaneous denitrification and metal recovery from Pb-Zn smelting wastewater was tested in BES inoculated with *Castellaniella* species. First, Cu and Hg were recovered in MFC mode attaching a 10 Ω external resistor, afterwards Pb and Zn were recovered in MEC mode by applying 1.0 V and 2.0 V, respectively (Amanze et al., 2023). Recovery and removal efficiencies of various metals applying microbial electrochemical technologies are summarized in Table 2. As shown in Table 2, high recovery efficiencies of 99% have been reached for the metals Cr, Cu, Ni and Zn. Metals have been either recovered with a MFC (e.g., Au(III)) or with a MEC [e.g., Fe(II)], depending if recovery took place spontaneously or an external energy was supplied. Furthermore, metal concentrations can vary widely between low concentrations (12 mg L^−1^) up to higher concentrations of 4000 mg L^−1^, which makes BES an interesting application for metal recovery from high metal burden bio-leachates as well as from heavy metal contaminated wastewater at lower concentrations.

**TABLE 2:** Removal/recovery efficiencies of various metals in bioelectrochemical systems.

Metal ions	Reactions	Redox potential vs. SHE	BES type	Metal concentration [mg l^-1^]	Removal/recovery efficiency [%]	References
Cr(VI)	Cr_2_O_7_^2-^ + 14H^+^ + 6e^−^ → 2Cr^3+^ + 7H_2_O	1.33 V	MFC	100	99.4	Wang et al. (2020)
Au(III)	Au^3+^ + 3e^−^ → Au^0^	1.50 V	MFC	500	74.2	Hubenova et al. (2023)
Ag(I)	Ag^+^ + e^−^ → Ag^0^	0.80 V	MFC	50 to 4000	98.2–92.3	Lim et al. (2015)
Cu(II)	Cu^2+^ + 2e^−^ → Cu^0^	0.34 V	MFC	1000	≥99.9	Heijne et al. (2010)
Pb(II)	Pb^2+^ + 2e^−^ → Pb^0^	−0.13 V	MEC	115	98.5	Zhang et al. (2020)
Ni(II)	Ni^2+^ + 2e^−^ → Ni^0^	−0.25 V	MEC	50–1000	33–99	Qin et al. (2012)
Co(II)	Co^2+^ + 2e^−^ → Co^0^	−0.28 V	MFC	40–60	96.4	Huang et al. (2019a)
Cd(II)	Cd^2+^ + 2e^−^ → Cd^0^	−0.40 V	MEC	12.26	50–67	Colantonio and Kim (2016)
Fe(II)	Fe^2+^ + 2e^−^ → Fe^0^	−0.45V	MEC	500	97	Luo et al. (2014)
Zn(II)	Zn^2+^ + 2e^−^ → Zn^0^	−0.76 V	MEC	91–413	45–99	Modin et al. (2017)

## 3 Metabolism-independent bio-recovery techniques

### 3.1 Biosorption

Biosorption involves a biological matrix which binds metal ions from aqueous solutions in a fast and reversible process (Yu et al., 2020). Compared to conventional adsorbents, biosorbents are usually more cost-effective and environmentally friendly while at the same time showing high sorption capacities for various metals (Golnaraghi Ghomi et al., 2020). Throughout the years, many biological materials have been evaluated as possible biosorbents next to biomass from microorganisms, such as plant-derived materials, biopolymers, agro-industrial waste, sludges, or the combination thereof (Fomina and Gadd, 2014; Torres, 2020).

#### 3.1.1 Fundamentals and mechanisms behind biosorption

Biosorption is metabolically independent and hence a passive process that happens through ionic, chemical, or physical mechanisms. Physical adsorption is a result of electrostatic interactions or van der Waals forces while chemical mechanisms include ion exchange, complexation, chelation, precipitation, or reduction (Kanamarlapudi et al., 2018). In general, electrostatic interaction seem to dominate the mechanism of adsorption of metals onto biosorbents, being more likely than chemical reactions (Yu et al., 2020). Using dead biomass makes the biosorption process more straightforward and user-friendly (Singh et al., 2024).

Complexes formed by two or more species can be either mononuclear if the central position is occupied by only one metal atom or polynuclear when more than one metal ion is found in the center of the complex. The interaction between metal ion and ligands is of covalent nature. Coordination happens when the metal atom accepts an electron pair from the coordinating non-metal atom. This covalent coordination bond is mostly formed by = O, -NH_2_, -NH, -O-R, -S, -N =, -OH or = NOH coordinating groups (Kanamarlapudi et al., 2018). Takahashi et al. (2010) report a different complexing behavior of REE with functional groups such as carboxylates, phosphates, or amines on bacterial cell surfaces. REE were primarily bound by phosphate sites while heavy rare earth elements form complexes with a large coordination number and light or middle rare earth elements at lower coordination numbers. The authors resume that phosphates sites present more stable binding sites than carboxylate sites (Takahashi et al., 2010). As pH increases, the contribution of carboxylate surface complexes increases, possibly due to deprotonation of the phosphate environment (Breuker et al., 2020). When the metal ion is bound at more than one place at a time by a specific agent, referred to as chelant, to form a ring structure this complex is called a chelate. Chelates are usually more stable than simple complexes because of the multiple binding (Razzak et al., 2022; Yaashikaa et al., 2024). Ion exchange might be the most important concept in biosorption where the metal ions get exchanged by counter-ions from the biosorbents surface. Amino/imidazole groups are examples for anion exchangers whereas carboxyl groups work as cation exchangers (Kanamarlapudi et al., 2018). The exchange of cellular metal ions (K^+^, Mg^2+^, N^+^, Ca^+^) was described by several authors as evidence for the biosorption of heavy metals (Torres, 2020; Zinicovscaia et al., 2020). For instance, Sieber et al. (2024) reported the increase in Mg^2+^ in bio-leachate solutions after biosorption with spent brewer’s yeast indicating the displacement of cellular metal ions during the biosorption of heavy metal ions (Sieber et al., 2024). Metal ions can form insoluble inorganic metal precipitates with the functional groups on the surface of the biosorbents which can complicate further sorption or desorption (Fomina and Gadd, 2014). Reduction results in the growth of crystals when the metal ions interact with some functional groups such as carboxyl groups and get reduced (Kanamarlapudi et al., 2018). In a study by Park et al. (2005) several fungal biomasses were tested for their ability to reduce Cr(VI) to Cr(III) and hence remove Cr(VI) from aqueous solutions. *R. oryzae* showed high reduction efficiencies of Cr(VI) into Cr(III) within 48 h.

#### 3.1.2 Different biosorbents

Key factors determining the capacity of a biosorption process include the metal characteristics, the process parameters and the biosorbents properties (Gavrilescu, 2022). A careful selection of the biosorbents is important since the surface area, chemical composition and porosity have a substantial impact on the adsorption efficiency (Yaashikaa et al., 2024). Most important functional groups include carboxyl, carbonyl, hydroxyl, phosphoryl, phosphate, sulfate, amide, amino and thioether groups (Gavrilescu, 2022). The capacities of a myriad of different biomass types have been investigated in thousands of research papers reporting different efficiencies within the same microbial species. Since the native biomass composition does not vary significantly between different species of the same genus, the biosorptive capacity of a biosorbent is mainly influenced by the reaction conditions and the pretreatment (Fomina and Gadd, 2014). Peptidoglycan carboxyl sites are described as the main binding sites for metal cations in gram-positive bacterial cells whereas phosphate groups (e.g., from teichoic acid or phospholipids) predominantly complex with metal ions in gram-negative species (Breuker et al., 2020; Gavrilescu, 2022). Moreover, the proteinaceous S-layer of bacterial cells and sheaths composed of polymeric materials including proteins and polysaccharides seem to be important in metal complexation (Gadd, 2009).

The cell wall of cyanobacteria consists as well of peptidoglycan as a major component and some species produce sheaths as well. Algal cell walls are more divers with cellulose being the only common component. In brown algae, for example, alginate plays a special role in the outer cell wall and is responsible for high metal uptakes mainly due to high amount of carboxyl groups (Fomina and Gadd, 2014; Castro et al., 2017).

Fungal cell walls on the other hand are complex macromolecular structures consisting mainly of chitin, mannans, proteins, and glucans but also other polysaccharides, lipids and pigments (Breuker et al., 2020). Phosphorylated mannose residues in the outer fungal cell wall generate negative charges making them available for the biosorption of metal cations (Gavrilescu, 2022). In addition, fungal phenolic polymers, and pigments such as melanin provide potential metal-binding sites with their carboxyl, carbonyl, phenolic and alcoholic hydroxyl and methoxy groups (Fomina and Gadd, 2014).

Most studies so far have been focusing more on improving the reaction conditions for various microorganisms than comparing the differences between the microorganisms. In an interesting study by Breuker et al. (2020) the biosorption efficiencies of different microorganisms of a mixed solution containing eight REEs at low pH was investigated. They could show a selective preference of the gram-positive bacterium *Bacillus subtilis* for the heavy REEs ytterbium and lutetium at pH 2.5 whereas the tested fungi (*Catenulostroma chromoblastomces* and *Pichia* sp.) showed a preference for middle REEs at the same conditions. Additionally, a better biosorption was achieved when using live *versus* dead biomass confirming cell integrity as an important parameter for biosorption.

#### 3.1.3 Parameters affecting biosorption

The possibly most important physico-chemical parameter influencing biosorption is the solution pH. The pH affects the charge of the functional groups on the biosorbent surface but also the speciation of the metal in solution. The predominant form of metals in aqueous solutions are cationic species. Therefore, a more negative charge of the biosorbents is beneficial for the biosorption of metals and the most suitable pH range for metal biosorption is between 7.0–8.0 (Torres, 2020). However, chemical species can also be anionic, exist as complexes or in a variety of oxidation states. Even more common metals in aqueous solutions such as Cu, Cd, and Zn can be complexed or hydroxylated depending on the pH or the composition of the solution. Nevertheless, it is often assumed that metals are solely present as divalent cations and metal speciation is ignored in many studies (Fomina and Gadd, 2014). Most biosorption studies so far have been conducted for one or two elements in solution and only at mild acidic conditions (pH > 3). At lower pH, the competition between protons and metal cations for the binding sites often results in a reduced biosorption of metals like Ni, Co, Zn, Cu and Cd (Breuker et al., 2020). When considering anionic metal species such as chromium, molybdenum, arsenic and others, a more acidic pH between 2.0–4.0 seems more favorable (Torres, 2020). Castro and co-workers (2017) studied the protonation-deprotonation of carboxyl groups on the surface of *Fucus vesiculosus* and sugar beet pulp as a function of pH. At pH values lower than the pKa of the different carboxylic groups the ligands of the cell wall are associated with hydrogen ions restricting the adsorption of metal ions. They report higher adsorption rates for Zn^2+^, Cu^2+^ and Cr^3+^ at pH 5-6 than at very acidic pH (Castro et al., 2017). Sieber et al. (2024) observed a similar trend with higher adsorption rates for Cu^2+^ and Zn^2+^ by increasing the solution pH up to 5 and 7.5, respectively, when using spent brewer’s yeast as a biosorbent (Sieber et al., 2024).

Pretreatment of biosorbents can contribute to an increased availability of metal-binding sites and hence, increase the adsorption capacity. The introduction of functional groups such as -NH_2_, -C=O, -COOH, and -OH contributes to metal sorption (Yu et al., 2020). Chemical treatment with organic solvents (Yaashikaa et al., 2021), alkaline (Mirmahdi et al., 2022) or acidic solutions as well as physical treatment including heat or fragmentation can change the surface of the biosorbents and at the same time increase the metal uptake (Yaashikaa et al., 2021). Chemical treatment of the brown algae *F. vesiculosus* with calcium chloride for instance increased the maximum sorption capacity for copper, lead and nickel. The authors of the study suggest that calcium that is retained by alginate in the cell wall of brown algae plays an important role in ion exchange (Rincón et al., 2005). For *Saccharomyces cerevisiae*, Göksungur and colleagues (2005) showed an improvement in Cd^2+^ and Pb^2+^ removal up to 31.75 mg g^−1^ and 60.24 mg g^−1^, respectively, after ethanol treatment of the cells. Ethanol treatment fixes soluble protein in the cell wall and hence increases the available metal binding sites (Göksungur et al., 2005). Temperature also has an influence on biosorption efficiencies, although this effect might be more evident when living biomass is used since the metabolic activity can increase with rising temperature (Torres, 2020). In addition, experimental conditions including biosorbents dose, metal concentrations and the contact time in the aqueous solutions influence the biosorption efficiency (Yaashikaa et al., 2021).

#### 3.1.4 Advantages, limitations and overcoming bottlenecks

Conventional methods such as chemical precipitation and electrochemical treatment produce a large quantity of sludge and are ineffective when treating aqueous solutions with metal concentrations among 1–100 mg L^−1^. Other methods including ion exchange, membrane technologies and activated carbon adsorption processes get very expensive when treating large volumes of wastewater containing heavy metals at low concentrations (Wang and Chen, 2006; Costa et al., 2021). Biosorption can offer a cheap and environmentally friendly alternative (Stathatou et al., 2022). It is evident that most biosorbents need to be modified to reach efficiencies comparable to commercial ones. As mentioned, there are several chemical or physical modifications that can be applied mainly to dead biomass (Yaashikaa et al., 2021). Immobilization of biomass by various matrices such as alginate (De Rossi et al., 2020), silica gel (Sayin et al., 2024) or on magnetic nanoparticles (Giese et al., 2020), can also improve the biosorption process. The immobilization increases the mechanical resistance of the biomass, facilitates the separation from the polluted solution and allows the application of biosorption at a bigger scale, e.g., by packing the biomass in fixed-bed columns (Kanamarlapudi et al., 2018). However, these modifications can result in higher costs of the final product and could reduce the eco-friendliness of the process. In that case, using living biomass without modification can be more efficient than dead biomass and many microorganisms such as microalgae or bacteria can be cultivated with high yields and low cost (Torres, 2020). Nevertheless, the application of nonliving microbial biomass has the advantage of not being affected by metal toxicity and some sorbents can be regenerated after desorption of the metal ions. Over time, fouling can block some metal-binding sites on the surface of dead biomass and hence it can lose its adsorptive properties (Gavrilescu, 2022). Most studies have meticulously investigated the biosorption process in batch experiments on a lab scale including kinetic measurements to determine adsorption isotherms. Fewer studies have moved to pilot plants and even less were implemented in industrial scale with real wastewaters (Staszak and Regel-Rosocka, 2024). Castro et al. (2017) performed a continuous biosorption in serial column reactor containing *F. vesiculosus* to clean wastewater coming from the electroplating industry. The system allowed the release of non-polluted waters for more than 6 h before saturation of the columns highlighting a possible industrial application. When testing the biosorption in a pilot plant with large glass columns (inner diameter of 7.5 cm and length of 100 cm) with a bed depth of 66 cm the process needed to be adapted to a mixture of the algal biomass with sugar beet pulp (Castro et al., 2017).

For the development of a sustainable adsorption procedure, the management of the spent sorbent is a crucial point. Up to a certain point, biosorbents can be regenerated and reused but eventually need to be disposed of. Up to date, this can be done either by incineration or landfill disposal (Harikishore Kumar Reddy et al., 2017). Metals can be leached from the spent biosorbents using strong acids or EDTA solutions. This desorption procedure needs a large amount of desorbing agent which will increase the cost of the sorption process and may cause secondary pollution. Liu et al. (2011) calculated the costs of recovering Pb from *T. angustifolia* biomass by leaching with HCl or EDTA was 0.19$ or 4.41$ per ton wastewater. When the biomass was pyrolyzed the cost decreased to 0.06$ per ton of wastewater. Pyrolysis proved to be techno-economical beneficial in that particular case (Liu et al., 2011). While the low cost and eco-friendliness of biosorption are advantageous, the relative low selectivity remains a challenge. Recent developments in biosorption focus on the selective recovery of metals of interest by using defined proteins from biomass or by directly engineering improved microbes and enzymes (Pollmann et al., 2018). A promising approach was demonstrated by Li et al. (2019) who created high-capacity bioadsorbents for Ni from wastewater by displaying specific nickel-binding peptides on the surface of *Saccharomyces* cerevisiae (Li et al., 2019). The identification of such specific metal-binding peptides is not trivial and described in more detail in Chapter 3.2.

#### 3.1.5 Application of biosorption in bio-leachates and liquid waste streams

Studies about metal recovery by biosorption from batch systems using synthetic solutions are abundant. Fewer researchers have studied the effect of real wastewaters on biosorption (Costa et al., 2020). Some examples can be found in Table 3. Sayin and coworkers (2022) entrapped fungal biomass of *Lactarius salmonicolor* in a silica gel matrix to study the removal of Mn^2+^ and Co^2+^ from a wastewater treatment unit of a factory in Turkey. Within batch experiments they showed that the immobilization significantly increased the biosorption efficiency. After successful batch experiments, they tested the biosorption in fixed-bed column experiments. Removal rates were 88.28% and 65.79% for Co^2+^ and 84.58% and 83.74% for Mn^2+^ in batch and column systems, respectively. In wastewater, containing both metal ions, the biosorption efficiency was slightly lower (Sayin et al., 2024).

**TABLE 3:** Removal/recovery efficiencies of various metals with biosorption processes.

Target metal	Source	Biosorbent	Modification	Max. recovery [mg g^-1^ or %]	pH	Regeneration	References
Zinc (Zn)	Electroplating industry	*F. vesiculosus*	Oven dried	61.5 mg g^-1^	5	HNO_3_, H_2_SO_4_, HCl	Castro et al. (2017)
		Sugar beet pulp		5.2 mg g^-1^			
Cobalt (Co)	Wastewater treatment unit	*L. salmonicolor*	Silica gel immobilized	118.6 mg g^-1^	6	Not tested	Sayin et al. (2024)
Manganese (Mn)				127.7 mg g^-1^			
Chromium (Cr)	Soil analysis laboratory wastewater	*S. cerevisiae*	Alginate bead composite	34.7 mg g^-1^	2	Not tested	De Rossi et al. (2020)
Lead (Pb)	Detergent industry wastewater	*Mucor* sp. NRCC6	pulverized	15.0 mg g^-1^	5.5	Not tested	El-Gendy et al. (2023)
Nickel (Ni)				9.0 mg g^-1^			
Zinc (Zn)				5.0 mg g^-1^			
Manganese (Mn)				2.8 mg g^-1^			
Aluminum (Al)	Waste printed circuit board leachate solution	*S. cerevisiae*	Lyophilized	5.3 mg g^-1^	3.5	Biogenic sulfuric acid	Sieber et al. (2024)
Copper (Cu)				4.2 mg g^-1^	5.0		
Zinc (Zn)				7.5 mg g^-1^	7.5		
Nickel (Ni)				0.78 mg g^-1^	8.5		
Copper (Cu)	Waste printed circuit board leachate solution	*Aspergillus oryzae*	None	88.6%	2	0.1 N HCl	Sinha et al. (2018)
		Baker’s Yeast	None	70.9%			

Metal recovery by the combination of bioleaching and biosorption has emerged as a promising technology yielding high metal recovery rates (Yu et al., 2020). Sinha and colleagues (2018) investigated the recovery of copper after bioleaching of waste printed circuit boards. The leach liquor was subjected to biosorption by dead biomass of *Aspergillus oryzae* and Baker’s Yeast. Both biosorbents showed good recovery rates of copper (>88% by using *A. oryzae* and >70% by using Baker’s yeast) from a mixture of various metals present in the leach liquor. After desorption and electrowinning, 92.7% Cu was recovered from the eluate with a purity of 95.2% which was further used as an antibacterial agent against *E. coli* demonstrating significant antibacterial activity (Sinha et al., 2018).

A study by Sieber et al. (2024) elucidated the importance of solution pH as a selectivity criterium for metal recovery. They investigated the biosorption behavior of spent brewer’s yeast for four different metals (Ni^+2^, Cu^2+^, Al^3+^ and Zn^2+^) after solubilization of these metals from printed circuit boards by bioleaching. High recovery rates could be achieved with the proposed stepwise biosorption process, especially for Cu^2+^ (>50%) and Zn^2+^ (>90%) at the corresponding pH. Additionally, the reusability of the yeast biosorbents was demonstrated in up to 5 cycles (Sieber et al., 2024).

### 3.2 Metal-binding peptides

Metal-binding peptides have emerged as a critically important area of investigation in biotechnology, garnering substantial scientific interest due to their potential for groundbreaking applications in environmental remediation, including biosorption, and resource recovery. Within the biotechnology sector, the design, engineering and formulation of these peptides are unlocking innovative prospects for the development of metal sensors, catalysts, and bespoke biomaterials (Bassan and Marchesan, 2023). Through rigorous analysis of peptide-metal interactions, researchers are unveiling novel strategies for the manipulation of metal binding peptides, which are applicable across a wide range of industrial and environmental contexts. Of particular significance is the role of metal-binding peptides in biosorption processes and biomining technologies, where their effectiveness in extracting metals from leachates and secondary raw material waste is increasingly recognized. Studies such as those by Malachowski et al. (2004) and Samuel et al. (2021) underline this potential. Mejare and Bulow (2001) and Matthews et al. (2008) deepen the discussion on the use of metal-binding proteins and peptides in bioremediation, phytoremediation, and bioinorganic interactions. Ye et al. (2022) (Ye et al., 2022) provide a computational and structural perspective on the analysis and characterization of metal-binding sites in proteins. Finally, Parker et al. (2012) and Reddy and Prasad (1990) investigate the interactions of metals with specific proteins such as glutathione S-transferases and amyloid precursor proteins, as well as the occurrence and structure of heavy metal-binding proteins and peptides in plants, algae, and fungi.

#### 3.2.1 Fundamentals and mechanisms of metal-binding peptides

Peptides consist of chains of amino acids whose side chains, containing functional groups, significantly influence their properties and metal-binding capabilities. The diverse functions and side chains of amino acids give peptides remarkable flexibility in binding metal ions. Oxygen (O) in hydroxyl (-OH) and carboxyl (-COOH) groups, nitrogen (N) in amino (-NH2) and imidazole groups, and sulfur (S) in thiol (-SH) groups play crucial roles in metal binding. Natural amino acids such as serine and threonine provide hydroxyl groups, aspartic acid and glutamic acid contain carboxyl groups, histidine contributes imidazole groups, and cysteine supplies thiol groups (Sauser and Shoshan, 2021). Histidine is particularly important with its imidazole side chain, as it can act as a bidentate ligand, coordinating metal ions with both nitrogen atoms. Cysteine, with its thiol group, plays a critical role in metal binding, while aspartic acid and glutamic acid can chelate metal ions with their carboxylic acid side chains. Serine can also participate in metal coordination through its hydroxyl group (Farkas and Sóvágó, 2012). The specific amino acids with functional side chains thus contribute to the structural and functional diversity of metal-binding peptides. Besides the ability to coordinate metals, other amino acids can induce significant conformational changes caused by complexation with metals. A series of studies have examined the influence of peptide side chains on metal binding. In 2009, Heinz et al. discovered that aromatic side chains such as tyrosine, methionine, and phenylalanine play an important role in chelate formation and binding to gold surfaces (Heinz et al., 2009). Kožíšek et al. (2008) focused on the design of metallopolypeptides and showed that the affinities of metal cations can be influenced with the help of unnatural amino acids by using specific metal ion chelating sites for the design of the peptides (Kožíšek et al., 2008). Székely et al. (2022) conducted structural analyses of metal-binding peptides and identified stable, specific structures as well as the role of side chains in fine-tuning the metal-binding ability of multihistidine peptides.

Aromatic side chains like phenylalanine significantly influence the conformation and structure of metal-peptide complexes (Hu et al., 1995). Also, histidine residues, due to their imidazole side chains, are crucial for metal binding and affect the conformation of metal-peptide complexes (Sovago and Osz, 2006; Murariu et al., 2019). The presence of hydroxyl-containing amino acids such as serine and threonine increases the binding affinity to metals like aluminum, indicating their role in interaction with metal surfaces (Zuo et al., 2005). Another important influencing factor is the specific positioning of amino acids in the peptide chain. DeSilva et al. found that cysteine and alanine also affect the specificity and nature of metal binding (DeSilva et al., 2002). The side chains of these amino acids significantly contribute to the binding properties and stability of metal-peptide complexes.

The understanding of peptide-metal interactions has significantly advanced through analytical techniques such as X-ray crystallography, nuclear magnetic resonance spectroscopy (NMR), and mass spectrometry. These methods enable researchers to decipher the structural details of peptide-metal complexes at the atomic level and provide valuable insights into specific coordination geometries and binding affinities.

The efficiency of metal binding by peptides is influenced by various parameters, including pH value, temperature, metal and peptide concentration, and the specific form of the metal. Studies such as those by Schwaminger et al. (2017) and Hughes et al. (2017) emphasize how environmental conditions like pH value and temperature affect the stability and configuration of peptide-metal complexes. The speciation of the metal also plays a crucial role, as different oxidation states or complex forms of the metal can have different affinities for the peptide binding sites (Ohata et al., 2019). The influences of metal and peptide concentration, the mobility of peptides, the reaction environment, and metal speciation on biosorption are complex and vary depending on the specific interactions and involved conditions. The concentration of both metal ions and peptides significantly affects the binding affinity and capacity (Stair and Holcombe, 2005). The arrangement of amino acids in the peptides can also affect metal binding. The mobility of peptides on surfaces can affect their interaction with metal ions (Hughes et al., 2017). These factors together determine the efficiency and selectivity of metal binding to peptides, which is crucial for applications from bioremediation to the development of functional materials. Natural configurations of peptides as well as synthetic modifications also play an important role in their performance by influencing metal binding properties. The inherent structure of peptides, including their amino acid composition and sequence, has a major influence on their ability to bind metals and on the stability of the complex under different conditions (DeSilva et al., 2002; Stair and Holcombe, 2005). Synthetic modifications of peptides can improve their stability and binding properties under various environmental conditions (Hughes et al., 2017; Luther and Boyle, 2024). Competing ions and competing chelators also play an important role in the biosorption of metals to peptides. In the presence of competing ions, the binding of metal ions to peptides can be affected. For example, the presence of high concentrations of calcium or magnesium ions can compete with heavy metal ions for binding sites on peptides, leading to reduced efficiency of metal binding. Furthermore, competing chelators such as organic acids or other ligands can affect the binding of metals to peptides by forming stronger complexes with the metal ions, thereby reducing the availability of binding sites on the peptides.

Metal-binding peptides often resemble in function and structure natural proteins and enzymes that occur in living organisms. A classic example is the binding of zinc ions by metallothionein, a cysteine-rich protein. In metallothionein, cysteine residues coordinate zinc ions, contributing to the protein’s ability to store and transport metal ions.

#### 3.2.2 Advantages, limitations, and overcoming bottlenecks

The use of metal-binding peptides for the recovery of precious metals from wastewater offers both advantages and limitations. Metal-binding peptides exhibit high selectivity for specific metals, which allows for targeted metal recovery (Lee et al., 2019; Y. K; Li et al., 2020). Such strategies are also compatible with resource sources that are either too complex in composition or too low in concentration to be processed with more traditional techniques. This means that such methods can also be applied to resource sources that are currently not even considered as such, such as industrial waste streams or low-quality ores (Pollmann et al., 2018). In addition, the use of metal-binding peptides offers environmental benefits by reducing the need for harsh chemicals and minimizing the generation of toxic byproducts (Braun et al., 2018). The specificity of these peptides also contributes to a more efficient and cost-effective metal recovery process. While engineered microorganisms and enzymatic systems provide effective solutions for metal recovery, metal-binding peptides surpass these technologies in key areas. Peptides are more robust under extreme environmental conditions and offer higher specificity for target metals, minimizing interference from competing ions. Additionally, peptides can be synthesized and immobilized on reusable supports, unlike microbial systems that often require cell destruction for metal recovery (Zhu et al., 2019). This reusability and adaptability make peptides a more efficient and sustainable option for large-scale applications. In biomining, metal-binding peptides can be used to extract specific metals such as gold, copper, or nickel from ores or waste materials. The flexibility and adaptability of the peptides make them valuable tools in the development of sustainable mining practices. Moreover, ongoing research in this area is exploring the potential of metal-binding peptides to enhance the efficiency and yield of metal recovery processes (Pollmann et al., 2018). Metal-binding peptides are developed using phage display technology, a method that enables the identification of peptides with specific binding affinities to metal ions, surfaces and particles (Smith and Petrenko, 1997). This technology involves creating a library of peptides displayed on the surface of bacteriophages, which are then exposed to a target metal. Peptides that bind to the metal are isolated and sequenced to identify the binding motifs. The process often begins with the construction of a random peptide library displayed on the coat proteins of the phage. These libraries are then subjected to biopanning against metal targets, such as metal ions immobilized on a substrate. Through successive rounds of selection, washing, and amplification, peptides with high affinity and specificity for the target metal are enriched (Braun et al., 2018). Isothermal titration calorimetry (ITC) and UV/VIS spectroscopy are among the techniques used to characterize the binding behavior of these peptides to specific metal ions and gain insights into their binding capacity, stoichiometry, and thermodynamic parameters (Matys et al., 2020; 2022). Additionally, the peptides can be engineered to improve their binding properties or introduce additional functionalities. This can include modifying the amino acid sequence, adding multiple repeats of the peptide, or creating fusion proteins with other functional domains. These metal-binding peptides find application in the development of novel biomaterials, where they can be used to recover metals from wastewater streams, develop biosensors for metal detection, or create new materials with tailored properties (Tamerler et al., 2006; So et al., 2009; Adams et al., 2013; Li et al., 2019).

Through tools of directed molecular evolution such as phage surface display, metal-binding peptides can be developed to bind various metal ions with high specificity, making them adaptable for a wide range of applications. Additionally, the binding properties of peptides can be tuned both by adjusting the selection parameters and by modifying their amino acid sequence, allowing for optimization of their binding properties for specific metals. Another advantage is their robustness, as peptides generally retain their functionality under a variety of environmental conditions, which is of great importance for industrial applications. However, there are also limitations and challenges associated with metal-binding peptides. The synthesis and application of these peptides can be costly, which could limit their practical use on an industrial scale. Additionally, there are concerns about their toxicity and stability in biological systems, which could affect their efficacy and safety in certain applications. Although peptides can selectively bind metals, the overall efficiency of biosorption and subsequent metal recovery can be challenging. Here, problems with peptides binding either too tight to a specific target to engineer a subsequent easy release and recover strategy, or biosorption occurs incomplete and less efficient (Taylor et al., 2021). To address these challenges, various strategies can be pursued. On the one hand, improvements in peptide design through advances in genetic engineering and molecular biology can facilitate the development of peptides with enhanced binding properties and stability. This involves the use of computer-aided tools to predict and optimize peptide sequences for specific metal targets. On the other hand, technological advances in peptide synthesis as well as biotechnological peptide production and screening technologies can reduce costs and improve the scalability of peptide-based metal recovery systems (Lederer et al., 2019). These include automated synthesis procedures and high-throughput screening methods that can accelerate the development and testing of effective peptides, such as subsequent analysis of selection pools through next-generation sequencing (Braun et al., 2020).

#### 3.2.3 Application of metal-binding peptides in bio-leachates and liquid waste streams

In various application areas, metal-binding peptides have been explored for novel applications, mainly focusing on environmental remediation, resource recovery, and nanotechnology. Metal-binding peptides are increasingly used in resource recovery, as they exhibit high selectivity and affinity for specific metals (Malachowski et al., 2004; Braun et al., 2018; Rahman et al., 2021). They have been developed to bind rare earth elements, heavy metal ions, and toxic metals and have proven effective in processes for metal recovery and detoxification. These peptides can be immobilized on solid carriers for use in metal binding, and their metal-binding properties can be evaluated using novel techniques (Malachowski et al., 2004; Schönberger et al., 2021). In the area of environmental remediation, they are used in the bioremediation of environmental heavy metals, demonstrating strong affinities for heavy metal ions that can be used to remove or recover these metals from polluted environments (Table 4) (Nian et al., 2010; Nguyen et al., 2013). In a published study, a new approach for metal recovery from aqueous solutions using bioengineering of surfaces was examined. Here, metal-binding peptides were used to functionalize the surfaces of fungal mycelia, thus improving metal recovery. This technique proved particularly effective in the treatment of bio-leachates and liquid waste streams (Urbina et al., 2019). For the functionalization of mycelial surfaces, the peptides that bind to metals were equipped with a chitin-binding domain to adhere to the chitin surfaces of fungi. These functionalized surfaces were then used to extract metals from aqueous solutions. Copper was used as a proof of principle. It was shown that the functionalized mycelial surfaces were able to effectively adsorb and bind copper from the solution. The mycelia treated with the functionalized peptides showed a significantly higher metal-binding rate compared to untreated mycelia. For example, the mycelium treated with peptides was able to remove up to 92% of the available copper from the solution after a 30-min incubation. With a longer incubation time, almost 100% of the copper was removed. These results underscore the potential of the functionalized mycelia for use in biological waste treatment and metal recovery. The use of fungal mycelia offers the advantage that they can grow on a wide range of biomasses and are suitable for large-scale application, making them a promising technology for metal recovery and the treatment of industrial wastewaters. These results highlight the considerable potential of biotechnologically functionalized surfaces for efficient metal recovery and the treatment of bio-leachates and liquid waste streams, which can be both environmentally friendly and cost-efficient (Urbina et al., 2019).

**TABLE 4:** Removal/recovery of various metals with metal-binding peptides.

Target Metal	Source Material	Peptide Sequence	Development Method	Engineering Method	Thermodynamic Data (K_D_, ΔH)	References
REE	Leachate solution low quality	AACGDYNADGWIEFEELACA	Derived from lanmodulin	Eight copies of dLBT (16 single LBTs) were fused in tandem to the C-terminus of OmpA	Tm K_D_ = 9.30e-6 ΔH not published	Martin et al. (2005), Park et al. (2016)
Copper (Cu)	Cu-rich solution from electronic waste	HNLGMNHVHNLGMNHVLQGNRPLVTQGCLQGNRPLVTQGC	Improvement of natural motifs by rational design	Functionalizing of mycelium surface with metal-binding peptides	Cu K_D_ = 3.73e-6 M ΔH not published	Urbina et al. (2019)
Nickel (Ni)	Pregnant solutions from (bio)hydrometallurgy metal extraction from copper mining	CNAKHHPRCGGG	Phage surface display against metal ions immobilized on sol-gel-coated glass fiber fabrics	Single clone binding studies to develop peptides that are able to distinguish different metals contained in leachates	Ni K_D_ = 1.04e-4 M ΔH = 1.87e-1 kJ mol^-1^ Co No interaction found	Matys et al. (2020), 2022
Cobalt (Co)		CTQMLGQLCGGG			Co K_D_ = 5.09e-6 M ΔH = 3.56e-1 kJ mol^-1^ Ni K_D_ = 7.69e-6 M ΔH = 3.56e-1 kJ mol^-1^	
Gallium (Ga)	Industrial wastewater from semiconductor industry	NYLPHQSSSPSR	Phage surface display against IDA-immobilized gallium under buffered condition	Immobilization of peptides on polystyrene beads to distinguish gallium from arsenic in recovery process	Ga K_D_ = 9.45e-5 M ΔH = −1.68e-2 kJ mol^-1^ As No interaction found	Schönberger et al. (2021), Taylor et al. (2021)
Lithium (Li)	Industrial battery waste solution	GPGNP	Computational modeling	Recombinant expression at *E. coli* surface displaying protein OmpA	Not published	Lynn and Kushick (1984), Jeong et al. (2024)

Matys et. al. presented a novel application of specific metal-binding peptides, which could be used for the treatment of bio-leachates and liquid waste streams. These peptides were identified using phage surface display and are characterized by high selectivity and binding capacity for specific metal ions such as cobalt and nickel (Matys et al., 2020). The peptides exhibit specific binding properties to certain metal ions. A nickel-binding peptide (CNAKHHPRCGGG) and a cobalt-binding peptide (CTQMLGQLCGGG) were highlighted, both of which have high selectivity for their target ions. The binding properties of the peptides were studied using UV/VIS spectroscopy and isothermal titration calorimetry (ITC). These techniques allow the evaluation of the binding strength and specificity of the peptides under various buffer conditions. The identified peptides could be used in the development of biotechnological processes for metal recovery from industrial wastewaters and bio-leachates. Their ability to specifically interact with metal ions makes them promising candidates for the purification of metal-contaminated waters. These results underscore the potential of specifically binding peptides in biotechnology, particularly for environmental applications where the efficient and selective removal of metal ions from complex solutions is required (Matys et al., 2022).

The publication by Schönberger et al. (2021) (Schönberger et al., 2021) describes the application of gallium-binding peptides for the sustainable treatment of industrial wastewater streams. In this context, specific peptides were identified using phage display technology, which can effectively bind gallium from polluted waters, particularly from the semiconductor industry. These peptides were immobilized on polystyrene beads to create a robust and reusable biosorptive material structure. Various gallium-binding peptides were investigated, determining their affinity by using ITC. The experiments revealed the competitive effect of acetate ions as well, otherwise influencing endothermic peptide-gallium interactions. The peptides were immobilized on polystyrene beads and proved to be extremely robust for the biosorption of gallium from an aqueous solution. The immobilized peptides were tested both in synthetic solutions and in real industrial wastewaters, effectively adsorbing gallium from these solutions. The performance of the immobilized peptides was also tested under various environmental conditions, demonstrating their specific binding of gallium, underscoring their suitability for the treatment of industrial waste streams. These results demonstrate the potential of specifically binding peptides in the biotechnological treatment of industrial wastewaters, which is particularly important for the recovery of valuable metals such as gallium (Schönberger et al., 2021).

In the study “Recovery of Rare Earth Elements from Low-Grade Feedstock Leachates Using Engineered Bacteria,” genetically modified *E. coli* bacteria were used to extract rare earth elements from leachate solutions of low quality (Park et al., 2016). The bacteria were modified to carry lanthanide-binding tags (LBTs) on their cell surface, which should improve the adsorption of rare earths. This method was tested with various leachate solutions, including residues from metal mines and rare earth deposits. The modified *E. coli* cells showed a 2- to 10-fold increased adsorption capacity for REEs compared to unmodified control cells. This was achieved through the specific binding ability of the LBTs on the cell surface. The genetically modified bacterial cells showed increased affinity for REEs compared to other metals such as copper, offering the possibility to selectively extract REEs from solutions with mixed metals. This technology provides a cost-effective and environmentally friendly method for REEs from waste products and leachate solutions, which is particularly important for the recycling and minimization of mining wastes. In summary, the study demonstrates the potential of bioengineering approaches to improve metal recovery from low-quality leachate solutions, especially for valuable REEs, which could be of great significance for technological applications and environmental protection measures (Park et al., 2016). Similarly, advancements in peptide engineering have demonstrated significant potential for REE recovery. Recent studies have identified engineered REE-binding peptides and proteins using advanced techniques such as phage display, rational design, and homology modeling. Theses peptides exhibit high specificity and binding affinity for REES, making them valuable in developing robust, reusable biosorptive materials (Ye et al., 2024). A notable application involves immobilization of these peptides on solid supports to enhance stability and reusability, enabling cost-effective and sustainable recovery processes. Compared to microbial systems, these engineered peptides offer high selectivity and operate under a broader range of environmental conditions, making them particularly promising for large-scale industrial applications. Moreover, their application significantly reduces energy consumption and chemical inputs relative to traditional hydrometallurgical methods while achieving high selectivity for REEs even in dilute or complex waste streams.

Together, these bioengineering approaches–utilizing both genetically modified microorganisms and advanced peptide engineering–demonstrate the potential to transform REE recovery technologies. They offer innovative, sustainable solutions for extracting valuable elements from waste streams while reducing environmental impacts and resource dependency.

### 3.3 Siderophores

Siderophores are a major family of iron-chelating agents that play a key role in bacterial iron homeostasis. They generally have a molecular weight between 200 and 2,000 Da and are characterized by a very strong affinity for ferric iron (Fe^3+^). They are produced and secreted by various microorganisms under iron-restricted conditions to scavenge iron from their environment. In parallel, bacteria express transporters at their cell surface that can capture back these chelators once they have chelated ferric iron (Schalk et al., 2012). All siderophores that have been discovered show strongest affinity and preference to Fe^3+^. In fact, the stability constants of most siderophore-Fe(III) complexes are very high and hence, are considered as the best ligands for ferric ions. The interesting part is that they have rich coordination chemistry beyond Fe(III) and complex with other metals with strong affinity. Being a microbial product, it gives the potential of large production by cost effective means. Moreover, there are approximately 500 different siderophores produced by various microorganisms (Hider and Kong, 2010). Therefore, due to their vast diversity in denticity, functional groups, and binding affinity, siderophores offer chelation of a wide range of metals efficiently.

Although all siderophores possess a high chelating nature towards iron, however, differences in functional groups cause different binding affinity to ferric iron in different environments. This is because, most of the microorganisms produce different types of siderophores in addition to one which is dominantly produced to combat iron deficiency in different environmental conditions. The role of secondary siderophores is not clear but with recent studies results, it can be stated that they are produced in lower iron restrictions or in different conditions as that of primary siderophore.

#### 3.3.1 Fundamentals and mechanisms of siderophores

There exist two mechanisms by which all siderophores are synthesized. These two mechanisms differ on the basis of enzymatic machinery involved – 1) Non-ribosomal peptide synthetase (NRPS) dependent system and 2) NRPS independent system (NIS). Many proteins involved in biosynthesis of siderophores are NRPS and hence most of the siderophores are termed as Non-ribosomal peptides (NRPs) (Barry and Challis, 2009). With advancement towards the development of potential biotechnological applications using siderophores, understanding their biosynthetic process becomes essential for obtaining commercially economical production.

NRPS dependent system involves multi-modular enzymes to produce siderophores with a peptide backbone containing non-proteinogenic amino acids. The order of the incorporated amino acids is determined by order of NRPS domains rather than RNA template. Main steps involved in siderophore synthesis using NRPS assembly lines are 1) monomer selection and activation, 2) chain elongation and 3) chain termination. Hence forth, NRPS assembly line compose of adenylation (A) subunit for monomer selection and activation, condensation (C) domain and peptide carrier protein (PCP) domain for chain elongation, and thioesterase (TE) domain for chain termination (Crosa and Walsh, 2002).

**FIGURE 3:**
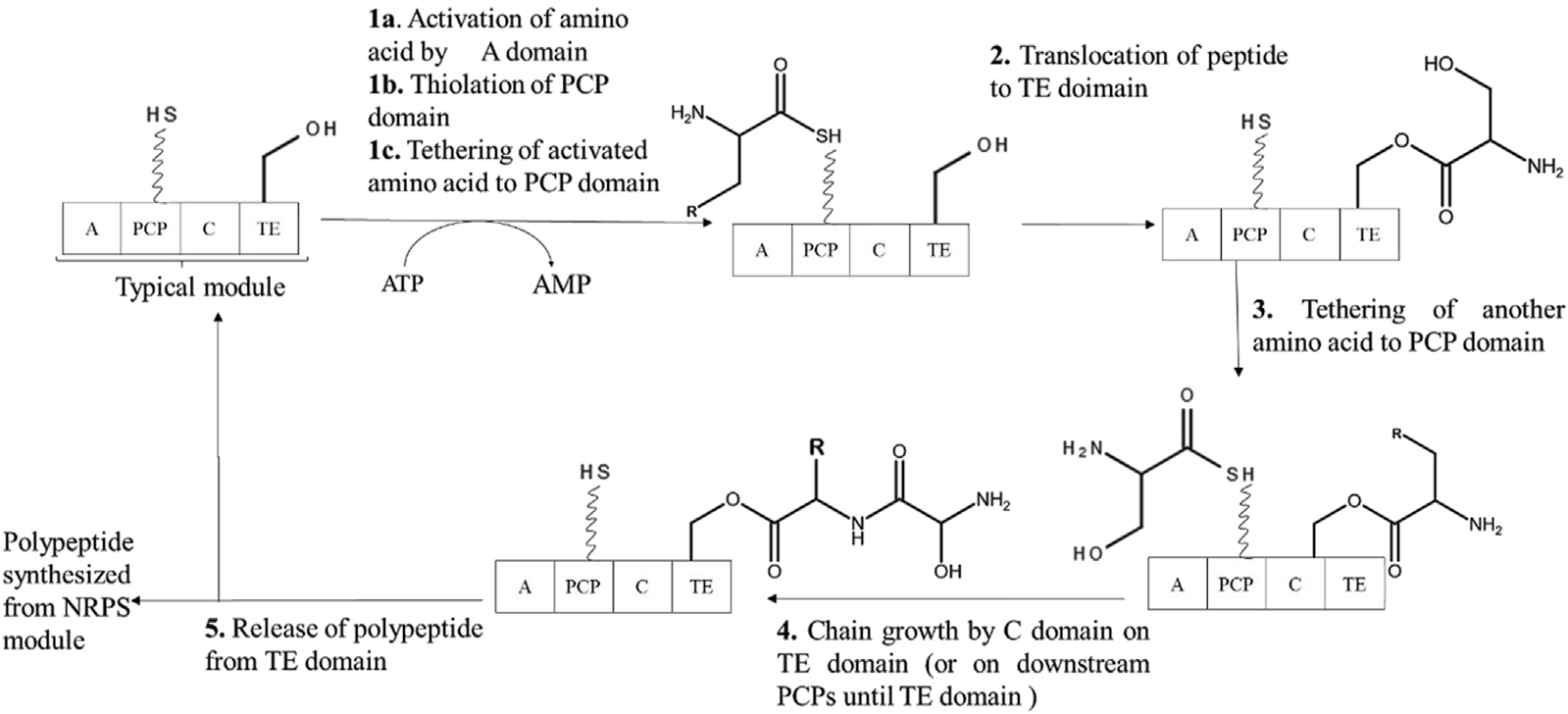
A representative typical NRPS module showing polypeptide synthesis. A: adenylation domain, PCP: peptide carrier protein, C: condensation domain, TE: thioeseterase domain.

Firstly, in the NRPS system, each PCP domain undergoes post-translation priming from apo to holo form by the enzyme phosphopantetheinyl transferase (PPTase). Holo form of PCP domain bears the phosphopantetheinyl arm which serves as a way station for chain growth. Following which, analogous to aminoacyl tRNA synthetase, A domain activates selected amino acid using ATP and then tethers the amino acyl moiety on the thiol way station of PCP domain. C domain, also known as peptide synthetase catalytic domain, catalyzes the peptide bond formation between the upstream peptidyl-S-PCP, which acts as a donor substrate, and proximal downstream aminoacyl-S-PCP, which acts as an acceptor substrate. This reaction grows the chain by one peptide and translocate the chain to the downstream PCP domain, which undergoes further elongation such that cascade of elongating acyl-S-enzyme leading to their formation. On reaching to the downstream PCP domain, the full-length chain undergoes chain termination which is catalyzed by TE domain. The chain is first transferred to the TE domain called as TE acylation. Depending on the TE domain it undergoes TE diacylation via intermolecular hydrolysis or intermolecular capture. In the cases, where TE domain is not present in the assembly line, growing acyl chains are transferred to soluble amine acceptors. In Figure 3, representation of such typical NRPS module for polypeptide synthesis in shown (Carroll and Moore, 2018).

**FIGURE 4:**
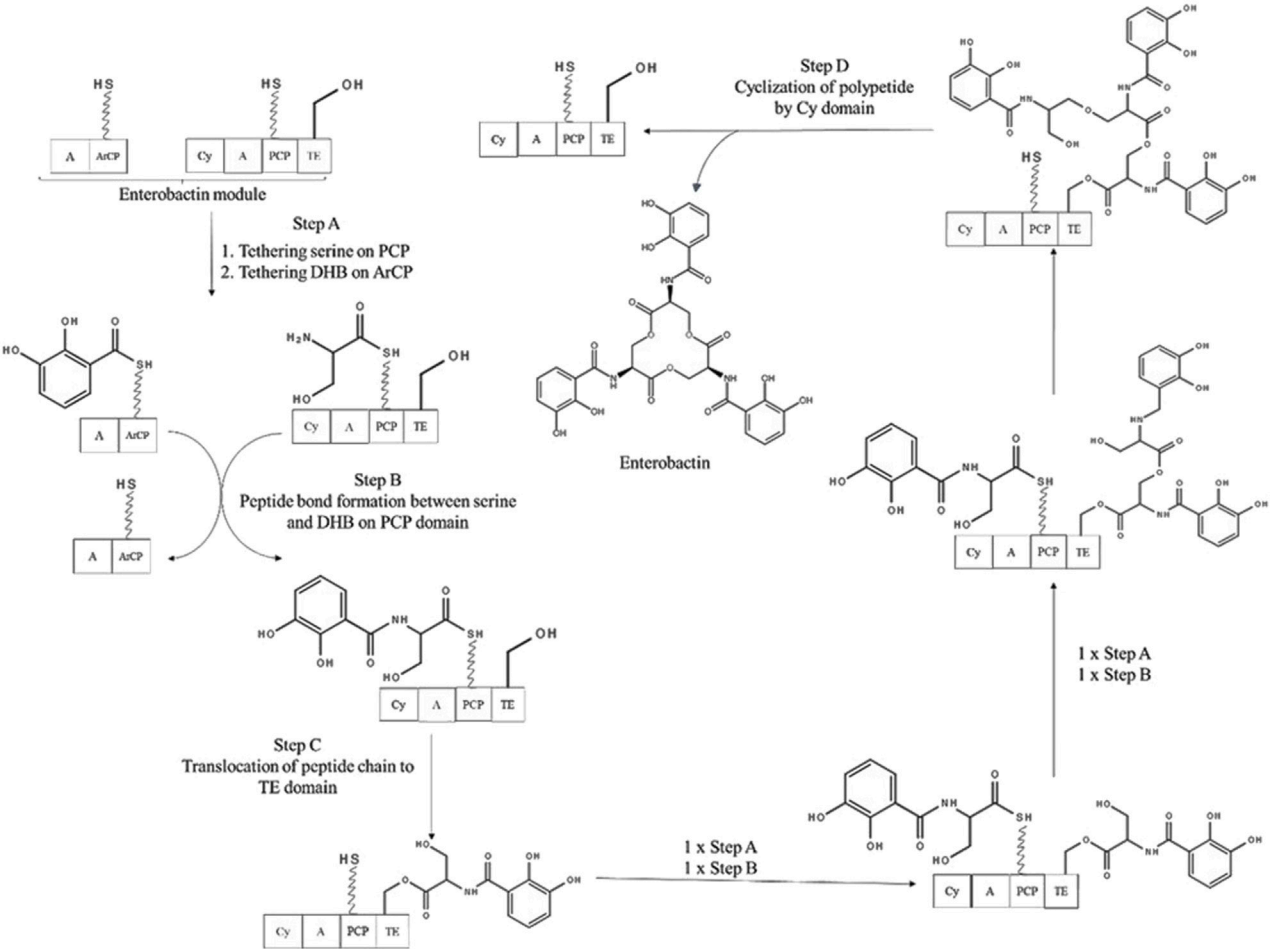
Enterobactin biosynthesis using NRPS module. Cy: Cyclization domain, A: adenylation domain, PCP: peptide carrier protein, C:condensation domain, TE: thioesterase domain, ArCP: Aryl carrier protein, DHB: 2,3-Dihydroxybenzoate (adapted from Walsh and Gary Marshall, 2014).

In addition to the basic NRPS assembly line, most bacterial species are identified to possess extra domains which are termed as Aryl carrier protein (ArCP) and Cyclization (Cy) domain (Zhou et al., 2007). ArCP performs a chain initiation step by installing a salicylic and 2,3 Dihydroxybenzoate (DHB) groups containing phenolic or catecholic moieties at the start of the assembly line. As shown in Figure 4, enterobactin biosynthesis using NRPS module consists of such additional domains. Firstly, a dedicated A domain catalyzes the formation of salicyl AMP or DHB-AMP and tethers it on the ArCP domain which is a subset of PCP domains. It then functions as the first donor in the chain elongation step. This additional step occurs in most bacterial species as it provides directionality on chain growth. Such siderophores contain aryl N-caps, which is analogous to N-terminal modification of the ribosomal protein biosynthesis. Cy domain catalyzes the formation of peptide linkage between the upstream donor substrate tethered in ArCP and downstream attacking substrate such as Cys-S-PCP, Ser-S-PCP, or Thr-S-PCP. This reaction is followed by side chain cyclization and dehydration which occurs when carbonyl group of peptide linkage is attacked by nucleophilic group (OH or SH) present in Cysteine, Serine or Threonine side chains. Following which, the chain is translocated to the next elongation module. Presence of Cy domains in NRPS assembly line provides the siderophore with thiazoline rings and oxazoline rings (Carroll and Moore, 2018).

The NRPSs consist of one megaenzyme which works in a coordinated fashion to link amino acids based on the order of its domain. However, NIS pathway machinery consists of multiple novel synthetase enzymes which function in an orderly fashion to produce a single siderophore product (Haynes and Challis, 2007). In general, the NIS pathway involves activation of citric acid or derivative via adenylation followed by condensation with a nucleophilic group typically amine or alcohol. The released citryl intermediate may be further condensed or macrocyclized to form dimeric or trimeric linear or cyclic siderophores (Challis, 2005). Different substrate specific NIS synthetase enzymes are involved to produce a diverse array of siderophores. Different types of NIS synthetases enzymes can be classified into three major types- Type A, B and C based on their substrate specificity. Type C’ as a subset of Type C has been recently introduced as a fourth classification of NIS enzyme which is based on phylogenetic analysis (Narh Mensah et al., 2023).

Type A enzymes such as SbnE are specific to citric acid and monoamine or amide substrates. It can be further subgrouped to Type A’ on the basis of the enantioselective nature of substrates and chirality of the final siderophore (Oves-Costales et al., 2009). SfnaB and SfnaD constitute Type A’ group which produces Staphyloferrin B and Staphyloferrin A respectively. Type B enzymes have substrate specificity to alpha-ketoglutarate and citryl-amine intermediates. Type C such as SbnF perform condensation reaction between monoamine or amide substrate with citryl-or succinyl-based intermediates (Cotton et al., 2009). The newly proposed further classification to Type C’ category has the unique ability to catalyze dimerization of citryl or succinyl intermediates followed by in some cases by macrocyclization to form cyclic siderophores (Oves-Costales et al., 2008; Carroll and Moore, 2018). DesD, AlcC, PubC, and IucC are involved in production of desferrioxamine, alcaligin, putrebactin, and aerobactin siderophores (de Lorenzo et al., 1986; Oves-Costales et al., 2009).

**FIGURE 5:**
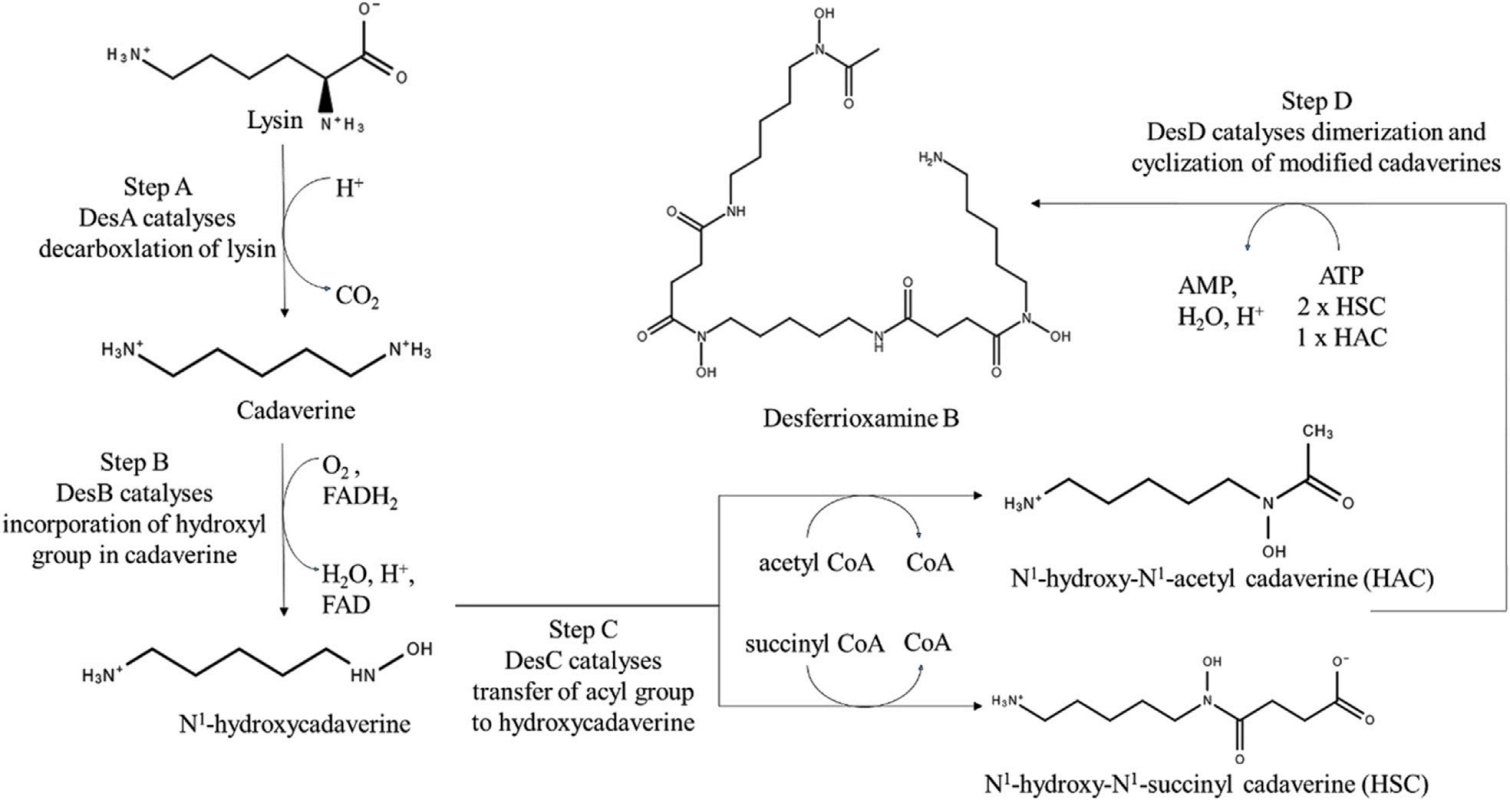
DFOB biosynthesis using NIS mechanism.

Desferrioxamine B (DFOB) is the most investigated siderophores for the metal complexation, thus looking at its synthesis is important. The synthesis involves the transformation of lysine through cascade of reaction catalyzed by enzymes DesA, B, C and D as described in Figure 5.

In addition to simple NRPS or NIS pathways, there exists a unique hybrid of NIS-NRPS pathways to produce siderophores in which unusual moieties are present because of the NRPS module and multiple rounds of amide bond formation takes place by a pair of NIS synthetases. This pathway produces “stealth siderophores” which tend to evade siderophore binding protein and gives organisms an advantage of virulence (Puja et al., 2023). An exemplified example of this pathway is petrobactin produced by *B. anthracis*. Petrobactin contains an unusual moiety 3,4- dihydroxybenzoate (3,4-DHB) which is thought to provide an ability for petrobactin to evade siderocalin binding (Koppisch et al., 2008). In addition to high affinity with iron, organisms have evolved to produce different kinds of siderophores to evade either siderophore capture or siderophore usage by other organisms in order to maintain iron supply for their metabolism. This suggests that siderophores play a significant role in iron acquisition.

#### 3.3.2 Siderophore coordination chemistry with iron

Chelation property of siderophores and their coordination chemistry with iron plays an important role for iron acquisition by microorganisms. Ferric complexation to siderophore is proton dependent. Stability of such complexes (Table 5) is dependent on the stoichiometry and hence on the pH of the solution. Among all siderophores, Enterobactin, a catechol siderophore having three catechol-amide units as bidentate chelator, has the highest known ferric stability constant (Karpishin et al., 1993; Hocking et al., 2010). Each unit loses two protons and distorts the metal coordination geometry from octahedral to near D3 symmetry. As compared to other functional groups in siderophores such as hydroxamate and carboxylate, catecholate orbitals have more overlap with the metal orbitals due to pi bond dominance contributing to high stability of the metal-ligand complex. In case of hydroxamate siderophores, ferric ion is chelated through adjacent N-O and carbonyl donors forming strong interactions. The energy of hydroxamate orbitals and metal orbitals does not match well due to which hydroxamates have 10 times lower formation constant as compared to catechols of same denticity (Hocking et al., 2010). Polydentate catechols have cyclic backbone which is connected via linker to catechol-amide groups projecting outwards. Whereas polydentate hydroxamates are connected in such a way that they form large rings. Therefore, on complexation with metals, polydentate hydroxamates form large chelate rings which do not provide additional stability, and hence lack the chelate effect (Winkelmann, 1991). Moreover, protonation of ferric hydroxamates occurs at lower pH (below 2) than ferric catechols (Harris et al., 1979).

**TABLE 5:** Stability constant of different siderophores with their functional group, denticity and biosynthetic mechanism.

Name	Denticity	Type	Biosynthesis mechanism	Logß(Fe^3+^)	References
Enterobactin	6	catechol	NRPS	49.0	Harris et al. (1979)
Desferioxamine E	6	hydroxamate	NIS	32.5	Konetschny-Rapp et al. (1992)
Desferrioxamine B	6	hydroxamate	NIS	30.5	Anderegg et al. (1963)
Rhodotorulic acid	4	hydroxamate	NIS	31.2	Carrano and Raymond (1978)
Bisucaberin	4	hydroxamate	NIS	32.2	Hou et al. (1998)
Alcaligin	4	hydroxamate	NIS	32.4	Hou et al. (1998)
Ferrichrome A	6	hydroxamate	NRPS	32	Hou et al. (1998)
Ferrichrome	6	hydroxamate	NRPS	29.1	Hou et al. (1998)
coprogen	6	hydroxamate	NIS	30.2	Hou et al. (1998)
ferricrocin	6	hydroxamate	NRPS and NIS	30.4	Hou et al. (1998)
Pyoverdine	6	mixed	NRPS	30	Evers et al. (1989), Albrecht-Gary et al. (1994), Harrington et al. (2012)
Rhizoferrin	6	carboxylate	NIS	25.3	Harrington et al. (2012)
Bacillibactin	6	catcehol	NRPS	33.1	Dertz et al. (2006)
Aerobactin	6	mixed	NIS	23.3	Winkelmann (1991)
Amonobactin T	4	catechol	NRPS	34.5	Winkelmann (1991)
Chrysobactin	2	catechol	NRPS	17.3	Sandy and Butler (2011)

Additionally, in catechol, ferric ion is first coordinated with meta phenolate. Meta phenolate coordinated to ferric ion becomes the most basic site in ferric catechol complex. Protonation of meta phenolate anions induces a rotation about the amide bond such that iron is coordinated by amide carbonyl and ortho phenol. This is termed as shift from catechol mode to salicylate mode. In comparison ferric catecholate stability is higher than the ferric salicylate complexes even though the bond strengths are same (Abergel et al., 2006). Increased strain caused due to such rotation effects differently in different catechol siderophores according to their carbon network and hence lowering formation constant in different magnitudes. Such alternative binding modes are not present in hydroxamate siderophores. However, due to formation of large chelate rings, concentration effects serve as an advantage to polydentate hydroxamate siderophores (Winkelmann, 1991). Lower concentration of trihydroxamate siderophore is needed to chelate the same amount of iron as compared to di- or mono-hydroxamate siderophores (Carrano et al., 1979; Winkelmann, 1991).

In addition to the protonation constant, siderophore preorganization also contributes entropically to the formation constant of the ferric complex of siderophores. In the catechol functional group, ferric ion first coordinates with meta and ortho phenolates. Even though meta phenolates are more basic and ortho phenolates are more acidic in this coordination, each metal-O bond distance is nearly identical. This is because ortho phenolate anions are stabilized by hydrogen bonding with adjacent secondary amides, forming a planar 6 membered ring. This facilitates enterobactin to preorganise for chelation such that its backbone is unperturbed on metal binding (Karpishin et al., 1993). Enterobactin and bacillibactin are structurally related but have different preorganization effects on ferric formation constants. Bacillibactin, having a glycine spacer connecting backbone to the catechol-amide groups, has lower ferric formation constant of the siderophore as compared to enterobactin (Dertz et al., 2006). Preorganization is also observed in hydroxamate siderophores. In fact, the first siderophore that demonstrated preorganization for metal chelation was observed in alcaligin, a dihydroxamate cyclic siderophore. As compared to linear dihydroxamate siderophore, rhodotorulic acid, alcalgin has 32 times higher formatic constant for 1:1 metal siderophore complex. However, in the 2:3 complex, as alcaligin favors mono-bridged complexes, its cyclic structure is distorted. This nullifies the preorganization effect and results in a similar formation constant as rhodotorulic acid which favors tri-bridged complexes (Hou et al., 1998; Crumbliss, 2005).

#### 3.3.3 Advantages, limitations, and overcoming bottlenecks

Siderophores have been observed to have the extended capability to bind to other metal cations as well. Compared to other ligands, siderophores and non-ferrous complexes have high thermodynamic stability. Iron (III) and gallium (III) have similar ionic radius and thus their complexes with siderophores also share similar structure. As mentioned above, different parameters contribute to stabilization of an anionic form of siderophore, similarly siderophore complexes with different metals having different ionic radius are also stabilized. For example, hydrogen bond between ortho phenol and amide group of catechol in enterobactin which contribute in stabilization of ferric enterobactin is also observed in cases of complex of enterobactin with vanadium (IV), silicon (IV), titanium (IV), gallium (III) and germanium (IV) (Borgias et al., 1986). An example of hydroxamate siderophore, DFOB which binds to iron with binding constant of 10^30^ M^−1^ also forms stable complexes with Ga^3+^, Al^3+^, In^3+^ with formation constants between 10^20^ M^−1^ and 10^28^ M^−1^ with preference as follows: Fe^3+^>Ga^3+^>V^3+^>Al^3+^>Zn^2+^>In^3+^ (Evers et al., 1989; Schwabe et al., 2018). Putrebactin, a stealth siderophore, has been examined for its chelation property with Cr(V), Mo(VI), and Mn(III). Similarly, Bisucaberin has shown to form complex with Mo(VI). The preference of iron over other metal is showcased by every siderophore but the diversity of binding different metal cations varies with each of the siderophore. Pyochelin and putrebactin can bind to Vanadium (V) ion existing in both of its form, Vanadyl ion (V (IV)) and Vanadate ion (V(V)) (Soe et al., 2014; 2016; Codd et al., 2018). However, pyoverdine and enterobactin can only bind to vanadyl ion and DFOB binds with vanadate ion only. Furthermore, complexation of curium (III) with pyoverdine secreted by *Pseudomonas fluorescens* isolated from the granite rock aquifers, is reported to bind stronger than EDTA, hydroxide or carbonate complex with curium. Pyoverdine and Pyochelin bind to range of metal (Ag^+^, Al^3+^, Cd^2+^, Co^2+^, Cr^2+^, Cu^2+^, Eu^3+^, Ga^3+^, Hg^2+^, Mn^2+^, Ni^2+^, Pb^2+^, Sn^2+^, Tb^3+^, Ti^+^ and Zn^2+^). Desferrioxamine has shown the ability to form stable complexes with plutonium (V) which is structurally diverse as well as larger metal ions than iron. It has also shown the ability to form stable complex with Nb (V) at a wide range of pH (4–7) (Radchenko et al., 2014).

Even though there are literature evidences of complexation formation of siderophores with different metals, there is limited data on quantification of the formation constants of these complexes. Table 6 contains some of the formation constant values available in literature for complexation of siderophores with metals other than iron.

**TABLE 6:** Stability constant related to different siderophores metal complex.

Name	logß (M^n+^) or pM	References
Desferrioxamine B	Fe^2+^(25.9), Co^2+^ (27.1), Ni^2+^ (27.66), Cu^2+^ (33.10), Zn^2+^ (28.17), Pb^2+^ (29.70), Sn^2+^ (40.76), Cd (6.17), Mn^3+^(29.9), Al^3+^ (24.50), Ga^3+^ (28.17), In^3+^ (21.39), Bi^3+^ (34.4), La^3+^ (21.9), Yb^3+^ (27.0), Co^3+^ (37.5), V^4+^ (29.66), V^5+^ (28.74), Mo^6+^ (53.14)	Evers et al. (1989), Kiss and Farkas (1998), Duckworth et al. (2009), Harrington et al. (2012)
Coprogen	Mn^3+^ (28.1)	Harrington et al. (2012)
Pyoverdine	Cd^2+^ (7.49), Cu^2+^ (17.67), Mn^3+^ (35.4, 35.3)	Andrejević et al. (2023)
Rhizoferrin	Mn^3+^ (29.8)	Harrington et al. (2012)
Pyochelin	Cu^2+^ (14.90), Zn^2+^ (11.8)	Andrejević et al. (2023)
Desferrithiocin	Zn^2+^ (7.9), Cu (12.9)	Andrejević et al. (2023)
Citric acid	Cd^2+^ (6.02), Cu^2+^ (6.24)	Andrejević et al. (2023)

In nature, even though catechol siderophores show high formation constant, most organisms prefer hydroxamate siderophores to acquire iron (Crumbliss, 2005). This is mainly because the high formation constant of catechol siderophore becomes a challenge in the following step of iron removal for iron utilization. The most common strategy used by bacteria for iron removal is reducing ferric ion to ferrous ion as siderophores form less stable complexes with ferrous ions. Ferric siderophore reduction is facilitated in the presence of ferrous chelator with similar reduction potential (Mies et al., 2006). Ferric hydroxamate siderophores have reduction potential in the range of −0.3 to −0.4 V which is comparable to biological reductants such as flavoprotein, iron-sulfur proteins and NADH (Matzanke et al., 2004; Crumbliss, 2005; Miethke et al., 2011). However, reduction potential of ferric catechol siderophores is in a far lower range, for example, ferric enterobactin has −0.75 V, which does not match with common biological reductants (Cooper et al., 1978; Harris et al., 1979; Raymond, 1979). In such cases bacteria employ hydrolyzing ferric siderophore complexes such that by reducing denticity, reduction potential is reduced to a range of biological reductants. As this process requires another set of enzymes and energy, in order to facilitate iron recovery from siderophores in simpler steps, bacteria prefer hydroxamate siderophores to catechol siderophores. Another alternative is to lower the pH such that coordination environment of catechol siderophores is altered. Such alteration can bring the reduction potential of catecholate complexes within biological range (Abergel et al., 2006). This can be considered as a useful methodology in metal recovery applications where metal siderophores decomplexation plays an equally significant role as metal siderophore complexation.

#### 3.3.4 Application of siderophores in bio-leachates and liquid waste streams

**FIGURE 6:**
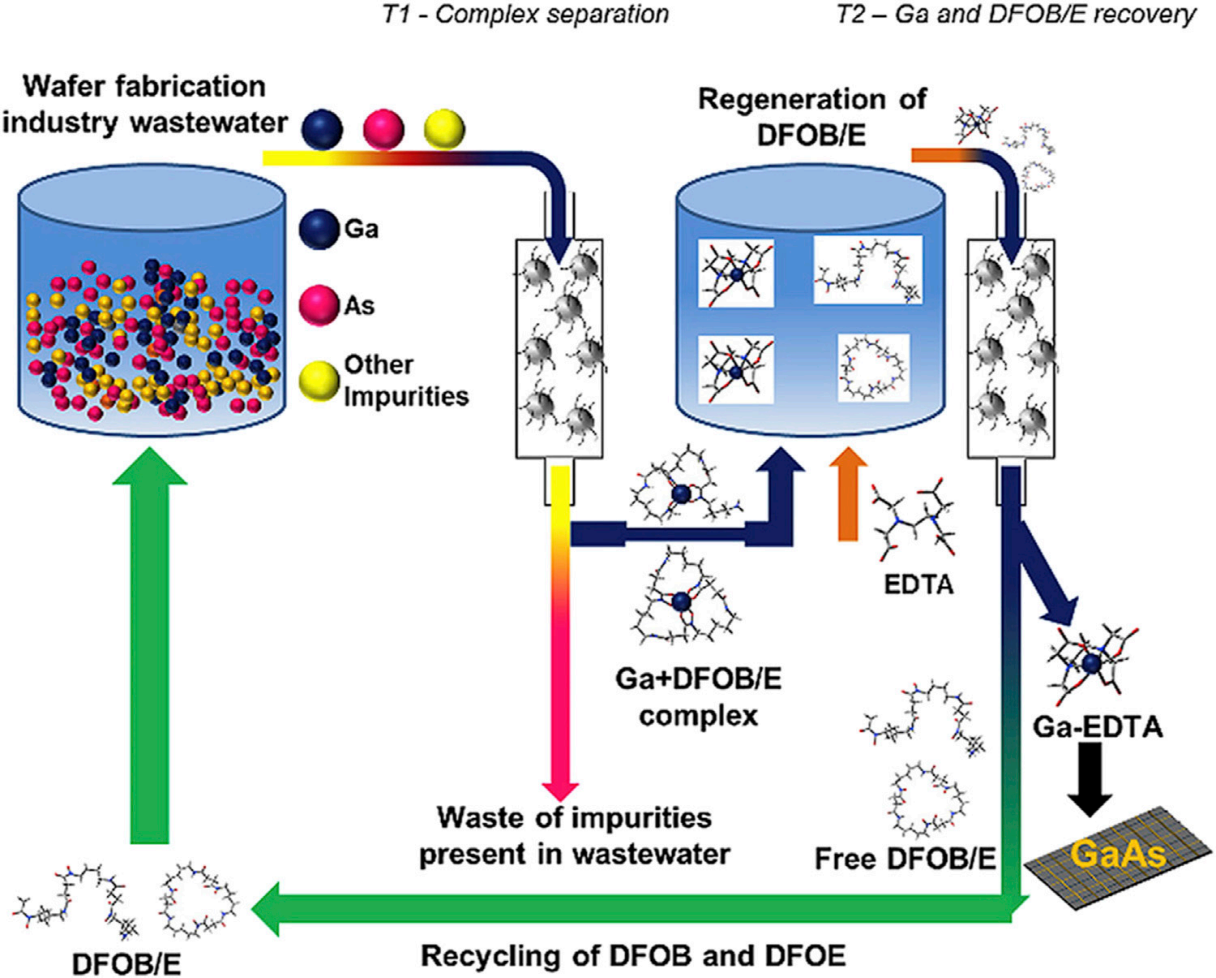
GaLIophore technology for recovery of gallium from wafer fabrication industry wastewater (Jain et al., 2019).

In case of metal recovery application, large pH change works for an advantage which cannot be done in living organisms. pH change and presence of synthetic ferrous chelator is an important step for metal siderophore decomplexation. GaLIophore technology (Figure 6), the first patented siderophore-assisted metal recovery technology, uses this method to recover critical and commercially important metals like Ga, Ge, In, etc. from wastewater from the fabrication industry which contains these metals in very low concentrations. In this technology, the wafer fabrication industry wastewater flows through reverse phase column and is incubated with siderophore solution. The flowthrough contains impurities which did not bind to siderophore. The elution fraction contains siderophore metal complex which then flows through the column for regeneration of siderophore. With the use of EDTA, siderophore is regenerated and metal is recovered with EDTA in complexed form. The EDTA-metal complex form can be further refined (Jain et al., 2019).

## 4 Conclusion and future perspectives

Nature offers a variety of tools, ranging from whole organisms to smaller microbial by-products such as siderophores or peptides that can be exploited for treatment of polymetallic waste streams. However, understanding the complexity of today’s waste streams and industrial byproducts is an essential prerequisite before considering appropriate treatment methods. So far, the application of bio-based technologies for the treatment of mixed metal solutions was mainly investigated for solutions of low concentration and complexity which is not the case for the majority of industrial effluents or bio-lixiviants. There are various reasons for this, for example, the toxic effects to the microorganisms at high metal concentrations or the higher costs involved in the production of metal-binding peptides leading to a higher economic burden of the technology. To date, a significant amount of work has been performed to tackle these challenges, e.g., implementing recombinant expression systems for small peptides or identifying new organisms with a higher tolerance for elevated metal concentrations.

Bioaccumulation still suffers from a poor understanding of the precise metal uptake mechanisms and the subsequent effect on the metabolism of the microorganisms. Future studies need to investigate the cellular mechanisms before moving to a scalable bioprocess level. The focus of biosorption studies has moved from lab scale to larger scale experiments paving the way for industrial application. Immobilization and modifications of the microorganisms are often necessary to achieve industrially relevant metal recovery systems. Recent insights into the fundamental principles of siderophores have been patented and demonstrate applicable technical solutions for selective metal recovery. The identification of peptide-based biomaterials for selective metal recovery has benefited from computational approaches in peptide engineering. It has clearly been shown that understanding the structure-function relationship of peptides is fundamental to develop robust and reusable biosorptive materials. Besides, BES profit from the incorporation within other hydrometallurgical technologies by improving metal extraction and recovery and the recent combination with machine learning techniques. Further research in this direction will promote the scale up and industrial application of BES for selective metal recovery.

Overall, bio-based recovery technologies offer promising opportunities for the treatment of complex liquid waste streams. By finetuning the parameters and modifying the presented tools, it is possible to selectively recover certain metals from mixed metal solutions. The combination of these different bio-based technologies allow for the selective recovery of critical metals from different waste streams in an environmentally friendly and sustainable manner.
